# Non‐Equilibrium Synthesis Methods to Create Metastable and High‐Entropy Nanomaterials

**DOI:** 10.1002/advs.202521953

**Published:** 2026-01-12

**Authors:** Shuo Liu, Chaochao Dun, Jeffrey J. Urban, Mark T. Swihart

**Affiliations:** ^1^ The Molecular Foundry Lawrence Berkeley National Laboratory Berkeley California USA; ^2^ Department of Chemical and Biological Engineering University at Buffalo The State University of New York Buffalo New York USA

**Keywords:** high‐entropy materials, metastable, nanomaterials, non‐equilibrium synthesis

## Abstract

Stabilizing multiple elements within a single phase enables the creation of advanced materials with exceptional properties arising from their complex composition. However, under equilibrium conditions, the Hume–Rothery rules impose strict limitations on solid‐state miscibility, restricting combinations of elements with mismatched crystal structures, atomic radii, valence states, or electronegativities. This severely narrows the accessible compositional space for creating new inorganic materials. In this review, we highlight how non‐equilibrium synthesis methods, featuring ultrafast heating and quenching, can overcome these thermodynamic barriers, enabling integration of immiscible elements into metastable and high‐entropy nanostructures. The resulting materials benefit from both kinetic trapping and stabilization by high configurational entropy, leading to enhanced phase stability. These materials can exhibit unique structural and functional properties that are needed for advancing catalysis, energy storage, thermoelectrics, and sensing. Furthermore, the ability of non‐equilibrium methods to generate unconventional compositions and structures expands the material design space dramatically, offering rich datasets for AI‐guided materials discovery. When combined with their inherent high‐throughput and scalable characteristics, these approaches enable rapid, iterative optimization and accelerate the development and industrial production of next‐generation inorganic materials.

## Graphical abstract

Synthesis of compositionally‐complex materials is limited by immiscibility of elements. Nonequilibrium synthesis methods reviewed here use ultrafast heating and cooling to overcome this limitation, creating metastable solid solutions and high‐entropy nanomaterials. This expands applications of inorganic materials in catalysis, electrochemistry, and sensors, while providing vast compositional space for material design.

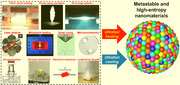

## 1 Introduction

Alloying, the oldest method of material development in human history, launched the transformative shift from the Stone Age to the Bronze Age. The discovery and utilization of bronze, an alloy of copper and tin, revolutionized agriculture, weaponry, and societal organization [1]. This approach has persisted through history and remains a cornerstone of modern materials development and optimization. From metals to ceramics, from bulk materials to low‐dimensional nanomaterials, and from structural materials to energy and functional materials, incorporating diverse elements into the crystal lattice of a host material, i.e., alloying or doping, can profoundly modify fundamental properties, such as crystal structure, electronic structure, defect density, mechanical strength, adsorption capacity, and thermal stability. These modifications often lead to significant performance improvements in specific applications. Examples range from conventional metals, where carbon incorporation into iron forms carbon steel with significantly enhanced strength and hardness [2]; to catalytic materials, where doping platinum‐based catalysts with cobalt or nickel boosts oxygen reduction reaction (ORR) activity and stability [3]; to photovoltaic materials, where metal doping in perovskite solar cells improves photoelectric conversion efficiency and long‐term stability [4]; and to adsorbents, where aluminum or zinc doping in metal–organic frameworks (MOFs) optimizes pore structures and surface activity, greatly enhancing CO_2_ capture capacity [5]. Traditional doping methods typically involve incorporating a small amount of a secondary component (often referred to as a dopant or solute) into the host crystal lattice, forming alloys, ceramic solid solutions, or multivariate MOFs to develop and optimize materials. While these solid solution materials have significantly contributed to human progress, traditional doping methods may not always be optimal. After centuries of development, most conventional alloy systems have been extensively studied and utilized, with certain performance parameters approaching their theoretical limits, leaving limited room for further innovation.

One way to overcome these limitations is by mixing multiple principal elements in relatively high (often equiatomic) concentrations. This concept forms the basis of high‐entropy materials, a class of materials characterized by combining five or more principal components in a single‐phase structure [6, 7]. High‐entropy materials can exhibit properties that are not observed in conventional solid solutions, such as entropic stabilization, sluggish diffusion, lattice distortion, and the so‐called cocktail effect, where the combined influence of multiple elements leads to synergistic enhancement in performance for a specific application. For example, atoms with varying radii occupying the same lattice sites induce significant lattice distortion, which increases atomic diffusion barriers and slows diffusion at elevated temperatures. This sluggish diffusion effect, combined with the entropy‐driven stabilization of the compositionally‐complex single‐phase structure, enhances the phase stability of high‐entropy materials. Distinctive characteristics of high‐entropy materials can include high thermal stability, enhanced mechanical properties, and superior corrosion and oxidation resistance. In recent years, greater research focus has been devoted to unique functional properties discovered in high‐entropy nanomaterials, such as outstanding catalytic activity, adsorption capacity, and electrochemical performance [8]. These features are driving extensive interest in high‐entropy nanomaterials for applications in catalysts, batteries, thermoelectrics, supercapacitors, hydrogen storage, solar cells, sensors, dielectrics, semiconductors, and magneto‐electrics [9, 10, 11].

For both binary and high‐entropy solid solutions, the thermodynamic immiscibility of components remains a significant challenge to practical synthesis. While an increase in configurational entropy can promote the formation of a single‐phase solid solution, macroscopic phase separation is still favored when the positive mixing enthalpy outweighs the stabilizing effect of the mixing entropy, preventing the system from reaching a stable or metastable homogeneous state. Therefore, conventional equilibrium‐based synthesis methods often rely on selecting elements with similar physicochemical properties. For example, RuRhPdIrPt high‐entropy alloy nanoparticles synthesized via co‐reduction feature Rh, Pd, Ir, and Pt, which all share an FCC crystal structure [12]; the (MgCoNiCuZn)O high‐entropy ceramic produced by ball milling and high‐temperature solid‐state reaction combines MgO, CoO, and NiO, all of which normally share a rock‐salt crystal structure [13]; and the MgCaSrBaMnFeCoNiZnCd multivariate MOF, prepared by solvothermal synthesis, retains the same M_3_O_3_(CO_2_)_3_​ building unit and MOF‐74 crystal structure of the constituent metals’ single‐component MOFs [14]. Recent studies have shown that elemental immiscibility can be overcome through non‐equilibrium synthesis approaches. For instance, a 15‐element FCC AuWCoNiPtRuMoPdRhFeCrIrSnMnCu high‐entropy alloy was synthesized via flash Joule heating [15], while the fluorite‐structured 22‐element (CeLaZrHfPrYGdDyNdErHoSmTbYbMgEuTmLuAlMnZnPd)O_2_ high‐entropy ceramic was produced by flame‐driven aerosol synthesis [16]. These examples incorporate immiscible elements from various regions and categories of the periodic table, offering an almost limitless compositional space for the development of novel inorganic materials.

Several recent reviews have provided important insights into high‐entropy materials from complementary perspectives. For example, George et al. [17] presented a foundational overview of bulk high‐entropy alloys, emphasizing equilibrium thermodynamics, phase stability, and mechanical behavior. Yao et al. [18] summarized high‐entropy nanoparticles with a focus on structure–property relationships, catalysis, and data‐driven discovery, providing only a brief overview of synthetic routes. Hsu et al. [19] clarified the four core effects of high‐entropy materials from a conceptual and theoretical standpoint. In addition, several other recent reviews have mainly classified existing synthesis methods for high‐entropy materials or nanoparticles and compared their features within conventional chemical, thermal, or near‐equilibrium frameworks, without developing a mechanistic understanding of ultrafast non‐equilibrium processes [20, 21, 22, 23]. These articles largely summarize established approaches method‐by‐method and do not address the kinetic factors that enable the formation of metastable or immiscible‐element solid solutions. In contrast to those prior reviews, we focus specifically on ultrafast non‐equilibrium synthesis routes and develop a unified kinetic framework explaining how rapid heating and quenching processes bypass equilibrium miscibility limits to generate metastable and multi‐element nanomaterials. Furthermore, we highlight the unique compatibility of these non‐equilibrium routes with high‐throughput synthesis, continuous aerosol production, and AI‐assisted discovery, offering a perspective distinct from existing reviews.

In response to the growing interest, inherent challenges, and immense opportunities in this field, this review aims to provide a comprehensive and forward‐looking perspective. We begin by considering the thermodynamic and kinetic principles governing the formation of metastable and high‐entropy solid solutions. Subsequently, we systematically review non‐equilibrium synthesis strategies that overcome thermodynamic limitations, highlighting the mechanisms of material formation and the resulting materials. Finally, we explore the transformative potential of these techniques in reshaping the landscape of inorganic nanomaterials, particularly in expanding the horizons of compositional and structural diversity and coupling with AI tools for accelerating the discovery of high‐performance materials.

## 2 Thermodynamic and Kinetic Principles

The formation of solid solutions arises from the interplay of atomic diffusion and thermodynamic driving forces. In alloys, ceramic solid solutions, or multivariate MOFs (containing multiple metal ions), atoms of different elements diffuse to occupy specific lattice sites, achieving a homogeneous distribution within the crystal structure. Diffusion is driven by gradients in the chemical potential of the diffusing elements. Moving down these gradients leads the system to minimize its Gibbs energy at a given temperature and pressure by either reaching the lowest‐Gibbs‐energy single phase or undergoing phase separation. However, diffusion and reaction pathways may lead the system through several metastable states. Desired metastable phases can be retained through kinetic control, as prototypically demonstrated in steel production, where quenching inhibits carbon atom diffusion, freezing the system into martensite or pearlite structures, which are desired based on their excellent mechanical properties. Extreme synthesis conditions trap structures that are stable at one set of conditions (e.g., at high temperature) under conditions where they are no longer the thermodynamically stable structure, for example facilitating the formation of supersaturated solid solutions [24]. Here, we use the NiO‐containing rock‐salt structured oxides as an example to illustrate the conditions for solid solution formation under equilibrium conditions, the role of configurational entropy, and how thermodynamic limitations can be overcome by rapid reaction kinetics.

### 2.1 Solid‐State Miscibility

Solid‐state mixing is governed by the Gibbs energy equation ΔG_mix_ = ΔH_mix—_T•ΔS_mix_. A thermodynamically stable solid solution forms when the Gibbs energy of mixing is negative (ΔG_mix_ < 0) at given T and P. Note that this must be satisfied not only for the pure components (e.g., elements or single‐cation oxides) but also for all combinations of phases into which the overall composition could split. Here, the mixing enthalpy (ΔH_mix_) reflects the energetic changes in interactions between elements upon mixing. If the interactions between dissimilar atomic species are less favorable than those between similar ones, ΔH_mix_ becomes positive, disfavoring mixing. Thus, a positive ΔH_mix_ indicates a thermodynamic driving force opposing solid solution formation, highlighting the immiscibility of the components. The Hume–Rothery rules, a set of heuristics initially developed to predict miscibility of metals, identify four factors that influence the mixing enthalpy: atomic radius, preferred valence state, electronegativity, and crystal structure [25, 26, 27]. Solid solutions form more easily when the atomic radius difference between elements is smaller, with a difference of less than 15% as the heuristic guideline form the Hume–Rothery rules. Larger differences cause lattice distortion as dopant atoms integrate into the host lattice, increasing elastic strain energy. Preferred valence state affects electronic compatibility. Forcing a solute atom to adopt a less stable oxidation state or to substantially donate or withdraw electron density from the solvent phase is generally energetically unfavorable. Electronegativity reflects an element's ability to attract electrons. When elements with significant differences in electronegativity are mixed, a pronounced redistribution of electron density occurs, leading to localized chemical bonds. This often leads to the formation of distinct chemical compounds (e.g., intermetallics or spinel structures) rather than substitutional solid solutions with random occupation of lattice sites. All these factors can significantly increase the mixing enthalpy. Crystal structure compatibility is the most crucial factor for solid solution formation. When different components have different stable crystal structures, there is an enthalpic cost for one of them to adopt the less‐favorable crystal structure of the other.

**FIGURE 1:**
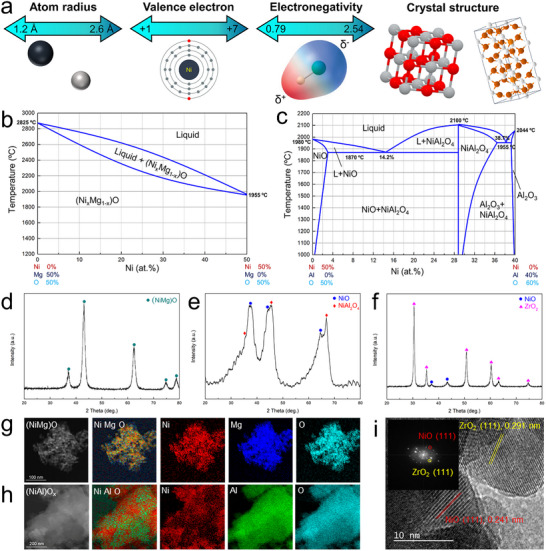
Solid‐state miscibility. (a) Illustration of the wide range of metal element characteristics that govern their miscibility as described by the Hume–Rothery rules; Phase diagrams of (b) NiO‐MgO and (c) NiO‐Al_2_O_3_ systems; XRD patterns of (d) NiO‐MgO, (e) NiO‐Al_2_O_3_, and (f) NiO‐ZrO_2_; Elemental maps of (g) NiO‐MgO and (h) NiO‐Al_2_O_3_, and HRTEM of (i) NiO‐ZrO_2_ material. The typical materials were fabricated by a near‐equilibrium co‐precipitation method with Ni:M = 20:80. Reproduced with permission [28]. Copyright 2024, Springer Nature.

Across the periodic table, elements exhibit significant differences in these properties (Figure 1a), which greatly limits the scope of solid solution materials that are thermodynamically stable at ambient conditions. For example [28], Ni and Mg have similar atomic radii and electronegativities, identical valence electrons, and their oxides, NiO and MgO, share the same rock‐salt crystal structure. Therefore, the binary phase diagram of NiO and MgO contains only one solid phase, indicating that they can form the (Ni*_x_* Mg_1‐_ *_x_*)O solid solution in any proportion (0< x < 1, Figure 1b). Thus, NiO‐MgO oxide prepared by the near‐equilibrium co‐precipitation at ambient conditions exhibits a single rock‐salt phase (Figure 1d) and homogeneous elemental distribution (Figure 1g). Ni and Al exhibit larger differences in these properties. In the binary phase diagram of NiO and Al_2_O_3_, three solid phases NiO, Al_2_O_3_, and NiAl_2_O_4_, are present, and phase separation dominates across most of the compositional range in the diagram (Figure 1c). Thus, NiO‐Al_2_O_3_ oxide synthesized under near‐equilibrium conditions shows separated NiO rock‐salt, NiAl_2_O_4_ spinel, and amorphous Al_2_O_3_ phases (Figure 1e) with inhomogeneous elemental distributions of Ni and Al (Figure 1h).

Moreover, the phase diagrams of NiO with most other oxides have not been reported, suggesting that NiO is immiscible with most metal oxides. Under equilibrium synthesis methods, it typically forms compounds such as NiFe_2_O_4_ spinel, LaNiO_3_ perovskite, and layered LiNiO_2_ oxides, or phase‐separated products, like NiO‐ZrO_2_ oxide prepared by co‐precipitation, which exhibited separate NiO and ZrO_2_ phases (Figure 1f,i). Nonetheless, the integration of diverse components into a single‐phase solid solution remains a cornerstone of advanced materials development. For example, the (NiMg)O solid solution is the precursor to one of the most potent catalysts for dry methane reforming [29]. Overcoming elemental immiscibility is not merely a fundamental challenge but holds the key to unlocking unprecedented opportunities in material innovation and performance.

### 2.2 Entropy‐Stabilized Solid Solutions

**FIGURE 2:**
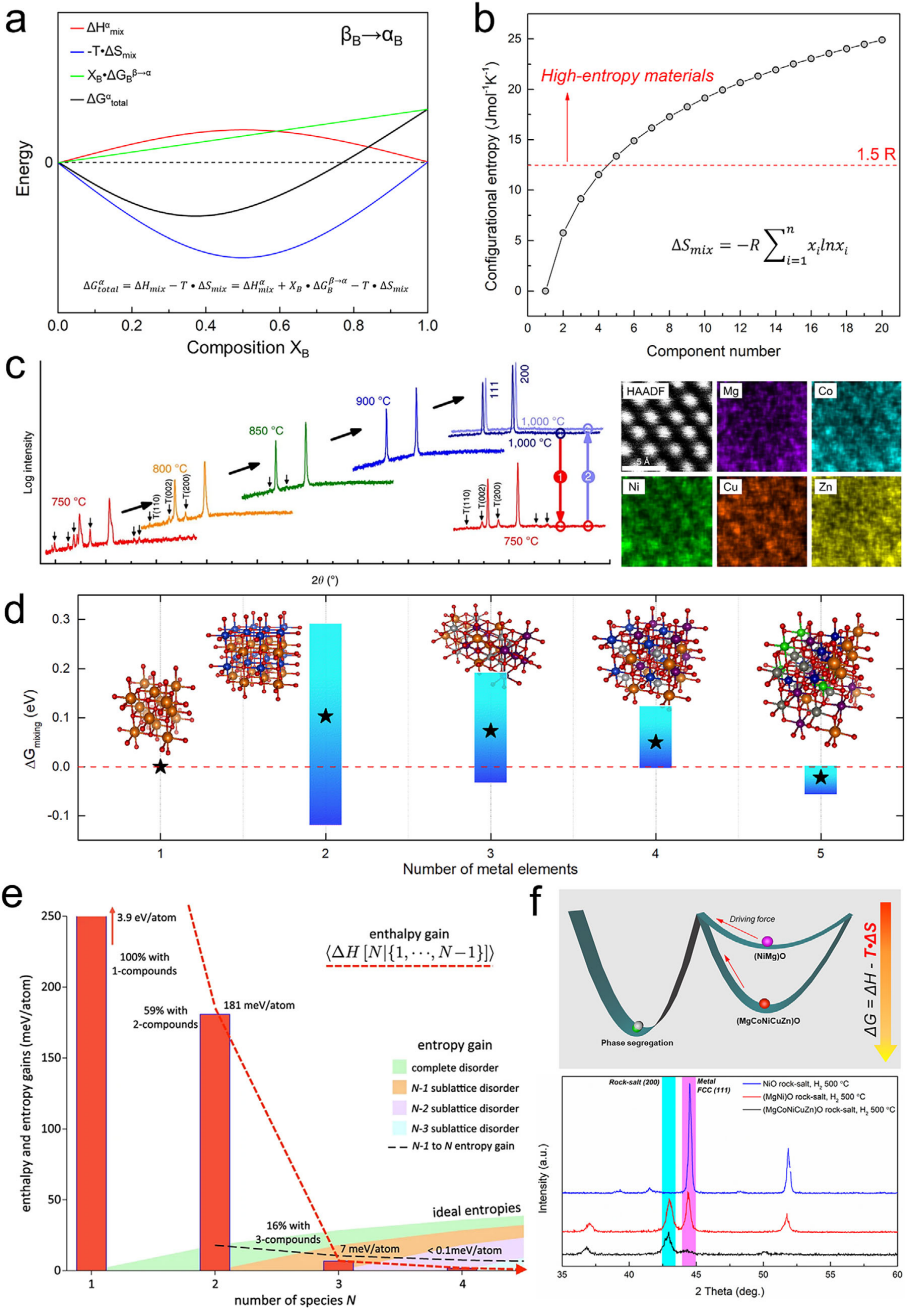
Entropy‐driven formation of solid solutions. (a) The regular solid solution Gibbs energy model of incorporating β phase component B solute into a α phase solvent to form an α phase solution, which qualitatively illustrates the competitive relationship between entropy and enthalpy when mixing non‐isostructural components; (b) Evolution of configurational entropy in an ideal solid solution; (c) XRD patterns obtained after calcining an equimolar mixture of MgO, NiO, ZnO, CuO and CoO at elevated temperatures for 2 h and corresponding elemental maps of (MgCoNiCuZn)O rock‐salt. Reproduced with permission [13]. Copyright 2015, Springer Nature; (d) Calculation of free energy changes upon mixing varied combinations of MgO, NiO, ZnO, CuO, and CoO into a rock‐salt lattice at 800°C. Reproduced with permission [16]. Copyright 2024, Cell Press; (e) Expectation of enthalpy‐gain and ideal entropy contributions as a function of number of species, *N*. Even at room temperature, entropy eventually overwhelms enthalpy in controlling phase stability of multi‐component systems. Reproduced with permission [34]. Copyright 2019, Springer Nature; (f) Schematic showing how configurational entropy increases the phase stability and XRD patterns of single NiO, binary (MgNi)O solid solution, and high‐entropy (MgCoNiCuZn)O rock‐salt oxides sintered in an H_2_ reducing atmosphere. Reproduced with permission [16]. Copyright 2024, Cell Press.

Under equilibrium reaction conditions, the key to dissolving non‐isostructural solutes and forming a stable solid solution is overcoming the positive enthalpy of missing of the components. The thermodynamic energy landscape involving a phase transformation is illustrated in Figure 2a, depicting the ideal mixing of a β‐phase solute B into an α‐phase solvent A [30]. The positive partial phase transition energy (X_B_•ΔG_B_^β→α^) is the barrier. For example, transforming ZnO with a wurtzite structure or CuO with a tenorite structure into a rock‐salt structure requires a phase transition energy of ΔG_ZnO_^wurtzite→rock‐salt^ = 24.26 kJ/mol at 1323 K for ZnO [31] and ΔG_CuO_^tenorite→rock‐salt^ = 22.2 kJ/mol at 1273 K for CuO [32]. Meanwhile, the negative term, −T•ΔS_mix_ can reduce the total Gibbs energy of the system. The configurational entropy (ΔS_mix_) is determined by the number and proportion of components in the solid solution, and a material is generally defined as high‐entropy when ΔS_mix_ exceeds 1.5R (Figure 2b) [18, 33]. Therefore, an increase in reaction temperature and configurational entropy can overcome the positive enthalpy of mixing, promoting the formation of a single‐phase solid solution. The most well‐known high‐entropy ceramic, rock‐salt structured (MgCoNiCuZn)O, was synthesized using this approach as reported by Rost et al. in 2015 [13]. The equimolar MgO (rock‐salt), CoO (rock‐salt), NiO (rock‐salt), CuO (tenorite), and ZnO (wurtzite) powders were uniformly mixed via ball milling, followed by high‐temperature solid‐state reaction to form the single‐phase rock‐salt structure. As the calcination temperature increased, the separated tenorite and wurtzite phases gradually disappeared, ultimately forming a homogeneous high‐entropy oxide (Figure 2c). Another study using DFT calculations demonstrated that at 800°C, the average ΔG_mix_ of the Mg, Co, Ni, Cu, Zn oxide system gradually decreases as the number of elements increases, reaching a negative value in the five‐component system (Figure 2d) [16]. Additionally, an increase in configurational entropy reduces the possibility of forming ordered compounds. Traditional binary material design typically aims to minimize enthalpy to achieve stable compounds. However, a large‐scale statistical analysis based on the AFLOW database examined the competition between enthalpy and entropy (Figure 2e) [34] and demonstrated that in quaternary and higher systems, entropic stabilization outweighs enthalpic effects, making materials more likely to form disordered solid solutions rather than ordered compounds.

From a structural perspective, the thermodynamic stabilization of a single‐phase multicomponent solid solution arises from both entropic and enthalpic contributions. For entropy, the dominant entropic term is often the ideal configurational entropy of random site occupation, $\Delta S_{config}=-R\sum_{i}x_{i}lnx_{i}$, which counters the tendency toward ordering or phase separation at finite temperature. Smaller, secondary entropy gains may also originate from disorder‐induced vibrational/defect entropy associated with lattice distortion, but these are distinct from the ideal mixing term. For enthalpy, in addition, two complementary enthalpic effects reduce the mixing enthalpy *ΔH_mix_*: (1) chemical averaging in a random alloy or ceramic solid solution creates nearly indistinguishable local environments that suppress short‐range ordering and diminish unfavorable unlike‐neighbor interactions; and (2) elastic/chemical relaxation of the sublattice accommodates size and bonding mismatches, lowering strain and unfavorable bond‐energy penalties. The overall stability window therefore, reflects the balance by $\Delta G_{mix}\approx \Delta H_{mix}^{chem+elastic}-T\left(\Delta S_{config}+\Delta S_{excess}\right)$, where Δ*S_excess_* denotes any non‐ideal entropy such as vibrational entropy and defect entropy. In this framing, lattice distortion and the “nearly indistinguishable local environments” are primarily enthalpic stabilizers, while configurational randomness provides the baseline entropic stabilization. The overall effect is the suppression of clustering and phase segregation, leading to localized heavy lattice distortion while maintaining overall homogeneity of composition [10, 30, 33]. This structure also provides kinetic stabilization, helping to retain a single phase even under conditions where it is only metastable (e.g., upon cooling). Atomic migration from one highly distorted lattice site to another requires greater energy (sluggish diffusion behavior) than in the corresponding ordered system [19]. Thermodynamically, the Gibbs energy of high‐entropy ceramics is lower than that of the comparable binary solid solutions, meaning that high‐entropy materials require a greater driving force for phase separation, i.e., the structure of high‐entropy materials is inherently more stable (Figure 2f). For example, using hydrogen reduction as the driving force, the NiO, binary (NiMg)O, and high‐entropy (MgCoNiCuZn)O rock‐salts were heated in flowing H_2_ at 500°C for 2 h. NiO was fully reduced to metallic Ni, while (NiMg)O underwent complete phase separation into metallic Ni and rock‐salt MgO. In contrast, the high‐entropy (MgCoNiCuZn)O largely maintained its rock‐salt structure, despite containing Co, Ni, and Cu, elements with low oxidation potentials whose individual oxides are typically reducible by H_2_ at temperatures below 300°C [16]. Importantly, entropic stabilization enables immiscible catalytic active sites (such as noble metals) to be incorporated into high‐entropy ceramic matrices and promotes their uniform, and even single‐atom, dispersion by increasing the configurational entropy of the system. Representative examples include single‐atom Pt incorporated into the (MgCoNiCuZn)O high‐entropy rock‐salt oxide [35] and Pd stabilized in the (CeZrHfTiLa)O_2_ high‐entropy fluorite oxide [36]. Once these active sites are trapped within the high‐entropy support lattice, the characteristic sluggish diffusion of high‐entropy materials places them in a lower‐energy metastable state (compared with their binary counterparts). As a result, a much larger driving force is required for these active atoms to ex‐solve and aggregate, thereby inhibiting sintering and enhancing the stability of the catalyst. In recent years, high‐entropy nanomaterials have produced groundbreaking advances in energy applications, particularly in catalysis [8, 37]. Their exceptional thermodynamic stability, surpassing that of conventional catalysts, allows them to withstand harsh reaction conditions while preserving structural integrity, making them highly attractive for next‐generation energy applications.

### 2.3 Overcoming Thermodynamic Limitations by Non‐Equilibrium Synthesis Methods

**FIGURE 3:**
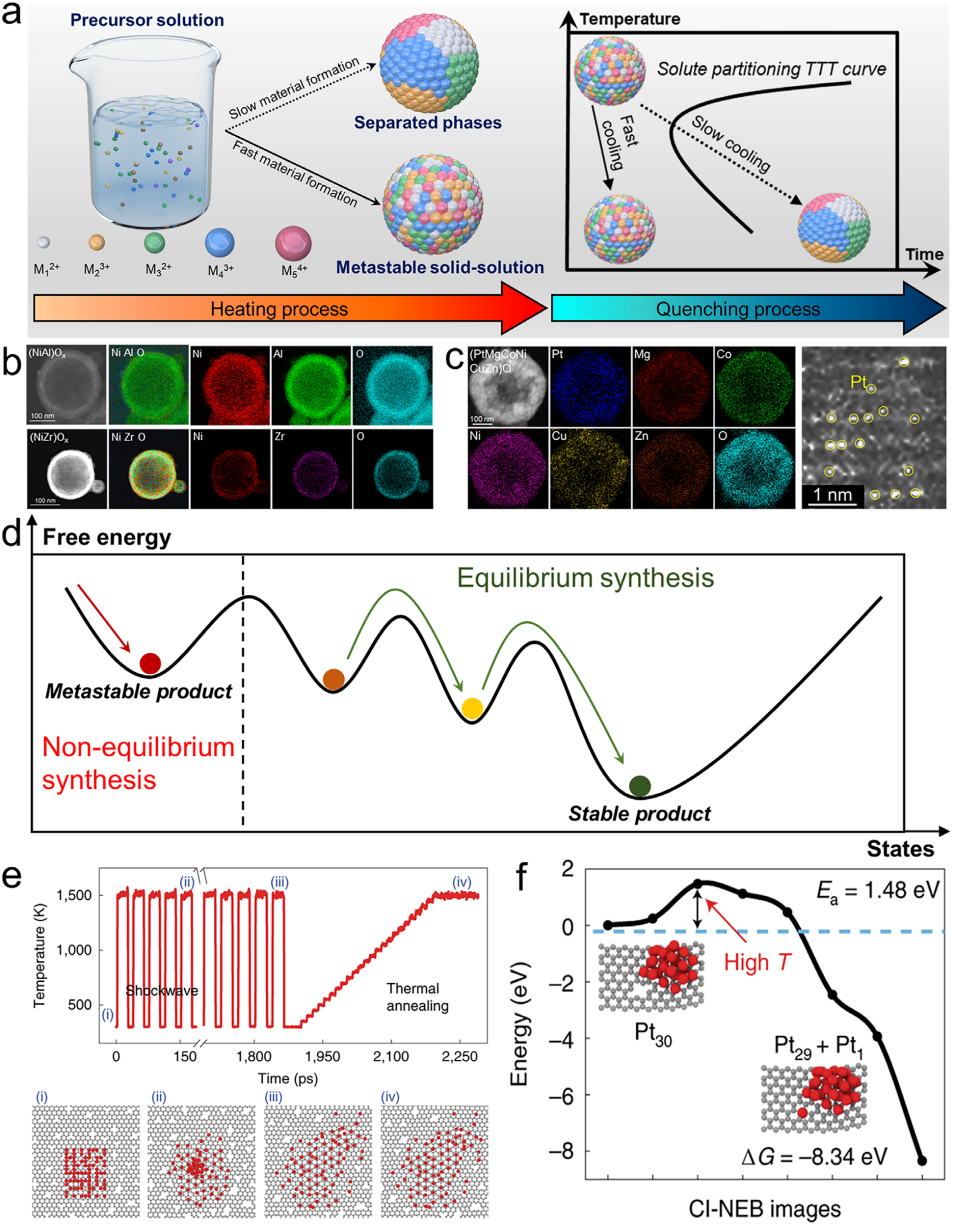
Non‐equilibrium synthesis routes for metastable and high‐entropy nanomaterials. (a) Schematic of mixing thermodynamically immiscible elements in a single‐phase solid solution, via rapid material formation followed by rapid quenching; (b) Elemental maps of immiscible NiO‐Al_2_O_3_, and NiO‐ZrO_2_ solid solutions. Reproduced with permission [28]. Copyright 2024, Springer Nature; (c) (PtMgCoNiCuZn)O high‐entropy rock‐salt synthesized by non‐equilibrium flame aerosol process. Reproduced with permission [16]. Copyright 2024, Cell Press; (d) Thermodynamic schematic of a metastable state product synthesized by a non‐equilibrium process vs. the stable state product synthesized by an equilibrium process. (e) MD simulation and (f) DFT calculation on the metastable single‐atom dispersion process in non‐equilibrium flash Joule heating. Reproduced with permission [44]. Copyright 2019, Springer Nature.

In addition to thermodynamic control through mixing enthalpy, mixing entropy, and temperature, kinetics also play a key role in determining the state of the material. For example, TiO_2_ prepared by low‐rate chemical vapor deposition tends to form the stable rutile phase [38], while high‐rate sputter deposition often favors the metastable anatase phase [39]. Consider an extreme scenario (Figure 3a) as follows. An aqueous solution contains multiple metal ions with significantly different atomic radii, valence states, and electronegativities, making them immiscible in the solid phase. However, in the solution, they are randomly distributed, achieving an ideal high‐entropy state. During slow heating and solvent evaporation, these metal ions will first form hydroxides and eventually transform into oxides. Governed by ΔG_mix_ = ΔH_mix_‐T•ΔS_mix_, the immiscible elements that were initially mixed form separated phases. However, if all the water were to evaporate instantaneously, the liquid‐to‐solid transition would occur before diffusion can take place. In this case, the random, homogeneous, and disordered distribution of metal atoms in the precursor would be directly transferred into the solid product. Then, immediate cooling, akin to quenching as often depicted using the Temperature‐Time‐Transition curve in metallurgy, can prevent phase‐separation, maintaining the high‐entropy, high‐enthalpy, and configurationally disordered state formed at high temperatures. In practice, the nucleation and growth cannot strictly occur instantaneously, but several practical methods can reach material formation timescales much shorter than the time required for diffusion‐driven phase separation, thereby overcoming thermodynamic limitations and incorporating immiscible elements into single‐phase solution solutions. For example, evaporation of aerosol microdroplets only takes a few milliseconds at sufficiently high temperature [40]. In this manner, the immiscible NiO‐Al_2_O_3_ and NiO‐ZrO_2_ systems, which undergo phase separation under equilibrium synthesis conditions (Figure 1), were successfully mixed into homogeneous solid solutions (Figure 3b) [28]. Similarly, a high amount of 10 wt.% noble metal Pt was mixed into a high‐entropy oxide lattice, forming (PtMgCoNiCuZn)O rock‐salt with single‐atom dispersion of Pt (Figure 3c) [16].

Metastability is fundamental to understanding why ultrafast non‐equilibrium synthesis can generate phases that lie beyond equilibrium predictions. Generally, a metastable phase is thermodynamically unfavorable but kinetically stabilized. It occupies a local minimum on the Gibbs energy landscape that is higher than the global minimum of the equilibrium phase but persists because a finite activation barrier (ΔG) prevents transformation. Turnbull famously classified metastability into three categories, including structural metastability, associated with non‐equilibrium crystal structures; morphological metastability, arising from defects or domain structures; and compositional metastability, in which solute concentrations exceed equilibrium solubility limits [41]. This metastable behavior is also fully consistent with the characteristics of materials produced by ultrafast non‐equilibrium synthesis. For example, non‐equilibrium routes tend to yield nanocrystalline or polycrystalline products rather than long‐range ordered single crystals [42]. Also, materials formed under such rapid conditions typically exhibit high defect densities [43], including cation vacancies, oxygen vacancies, and other non‐stoichiometric defects. In this review, however, the metastable phases of interest are primarily compositional metastable states, where multi‐element solid solutions form at concentrations far exceeding equilibrium solubility limits. This category directly corresponds to many multi‐component and high‐entropy materials obtained through non‐equilibrium processing, in which thermodynamically immiscible elements become homogenized within a single phase. This thermodynamic picture is schematically illustrated in Figure 3d. Under conventional equilibrium synthesis, slow heating and cooling allow the system to undergo sequential nucleation and phase transformations, enabling it to escape local minima of several metastable states and ultimately evolve toward the global Gibbs energy minimum, which corresponds to the most stable product. In contrast, ultrafast non‐equilibrium synthesis drives the system rapidly across the free‐energy landscape and traps it in a higher‐energy local minimum before diffusion and phase separation can occur. As a result, the material solidifies into a metastable phase that is thermodynamically unfavorable but kinetically persistent. Therefore, during ultrafast thermal processing, the precursor experiences a transient high‐temperature regime in which atomic mobilities increase dramatically and multi‐element mixing becomes possible, even for combinations that are immiscible under equilibrium conditions. Then, the high‐temperature mixed state is quenched directly into a metastable solid solution, without phase transition happening. This non‐equilibrium route contrasts sharply with the slow diffusion, compositional partitioning, and phase separation typical of conventional thermal synthesis.

Beyond the thermodynamic definition of metastability, molecular dynamics (MD) and first‐principles studies have provided important mechanistic insight into how ultrafast non‐equilibrium synthesis creates metastable structures at the atomic scale. Under shock‐heating or flash‐heating conditions, the precursor undergoes a brief high‐temperature excursion in which metallic clusters or partially ordered domains are driven into highly dispersed atomic configurations [44]. Molecular dynamics simulations have shown that, within just a few picoseconds, multi‐atom clusters can dissociate into individual atoms that become uniformly dispersed throughout a transient high‐temperature matrix (Figure 3e). The non‐equilibrium shock‐heating cycles disrupt Pt–Pt metallic bonding, promote rapid atomic diffusion, and facilitate the redistribution of atoms to defect‐rich sites, generating a highly mixed state that would not be accessible under equilibrium thermal conditions. Similarly, MD simulations of the flame spray pyrolysis process have also shown that the rapid, non‐equilibrium reaction environment can incorporate immiscible Pt atoms into the TiO_2_ lattice and achieve uniform single‐atom dispersion [45]. Meanwhile, first‐principles calculations further reveal the energetic pathway underlying this process (Figure 3f). Breaking a Pt–Pt bond to form isolated Pt atoms requires a substantial activation energy (Ea≈1.48 eV), which can be overcome only under extreme, ultrafast heating. Once liberated, these atoms can form strongly bound Pt–C configurations with a large exothermic stabilization energy (ΔG≈–8.34 eV). The combination of rapid heating, which activates otherwise inaccessible bond‐breaking events, and rapid quenching, which freezes the newly formed bonds before re‐aggregation can occur, results in the stabilization of metastable coordination environments rather than the thermodynamically favored Pt–Pt metallic state. These MD‐ and DFT‐based insights demonstrate that ultrafast non‐equilibrium routes access unique nucleation and growth pathways that fundamentally differ from equilibrium synthesis, enabling the formation of metastable, compositionally mixed nanostructures.

In addition, although ultrafast heating and quenching methods are generally described as globally non‐equilibrium processes, this does not exclude the possibility of forming brief, localized quasi‐equilibrium states during synthesis. Under extremely high heating rates, such as those found in flash sintering or laser melting, thermal diffusion at the nanoscale can be fast enough for small regions, including molten pools, reaction fronts, or gas‐solid interfaces, to temporarily reach local thermal equilibrium even when the overall system remains far from global equilibrium. Here, local equilibrium refers to a short‐lived state in which temperature and chemical potential become nearly uniform within a confined region over millisecond timescales, allowing short‐range atomic rearrangements to proceed in a quasi‐equilibrium fashion. These localized quasi‐equilibrium states do not contradict the non‐equilibrium character of ultrafast synthesis. Instead, they can be crucial for enabling rapid nucleation, short‐range homogenization, and kinetically trapped mixing before long‐range diffusion becomes significant. By allowing limited atomic rearrangement within a narrow time window, followed by immediate quenching that freezes the structure, such processes can effectively bypass global thermodynamic constraints. For example, recently reported programmable heating/quenching flash Joule heating clearly illustrates this principle [46]. In this method, the gas‐phase and adsorbed species closely follow the programmed temperature profile, and the applied current precisely sets the peak temperature, heating duration (0.02–0.1 s), and cooling rate. This temporal control allows local thermal equilibration to be initiated and then terminated with sub‐millisecond precision, creating reaction windows shorter than typical diffusion or sintering timescales. As a result, the system can selectively activate bond breaking, promote transient mixing, or induce partial melting, while quenching prevents processes such as grain growth, noble‐metal segregation, or transformation to equilibrium phases. The ability to controllably create and arrest such localized quasi‐equilibrium states offers important advantages for materials synthesis. It provides access to metastable or high‐entropy phases that need partial atomic homogenization but cannot tolerate extended diffusion. It also enables the stabilization of short‐range structures, defect configurations, and high‐entropy mixing states that would not persist under continuous heating. More broadly, this decoupling of activation from equilibration expands the range of achievable compositions and structures well beyond the limits imposed by conventional thermodynamic processing.

Here, we define non‐equilibrium synthesis methods as those that overcome the thermodynamic immiscibility of elements, transferring their disordered distribution in the precursor into a homogeneous solid solution nanomaterial. These methods often involve ultrafast heating (material formation) and cooling processes. Based on this definition, non‐equilibrium synthesis methods employ a bottom‐up strategy, creating a material from precursors of the elements in a different state (e.g., gaseous atoms or ions, or ions in solution). Many top‐down strategies, such as ball milling [47], exfoliation [48], dealloying [49], and physical vapor sputtering [50], which break down or reconstruct existing bulk high‐entropy materials into high‐entropy nanomaterials, are not the main focus of this review, even though they involve non‐equilibrium processes. Also, this review focuses on nanomaterials for energy and functional applications, so the synthesis methods for bulk structural materials, such as the most commonly used vacuum arc melting method for bulk high‐entropy alloys [51], are not included. Additionally, some equilibrium synthesis methods that can incorporate immiscible elements, such as lowering the mixing enthalpy by utilizing low‐melting‐point metals [52, 53], are also outside the scope of this review.

**FIGURE 4:**
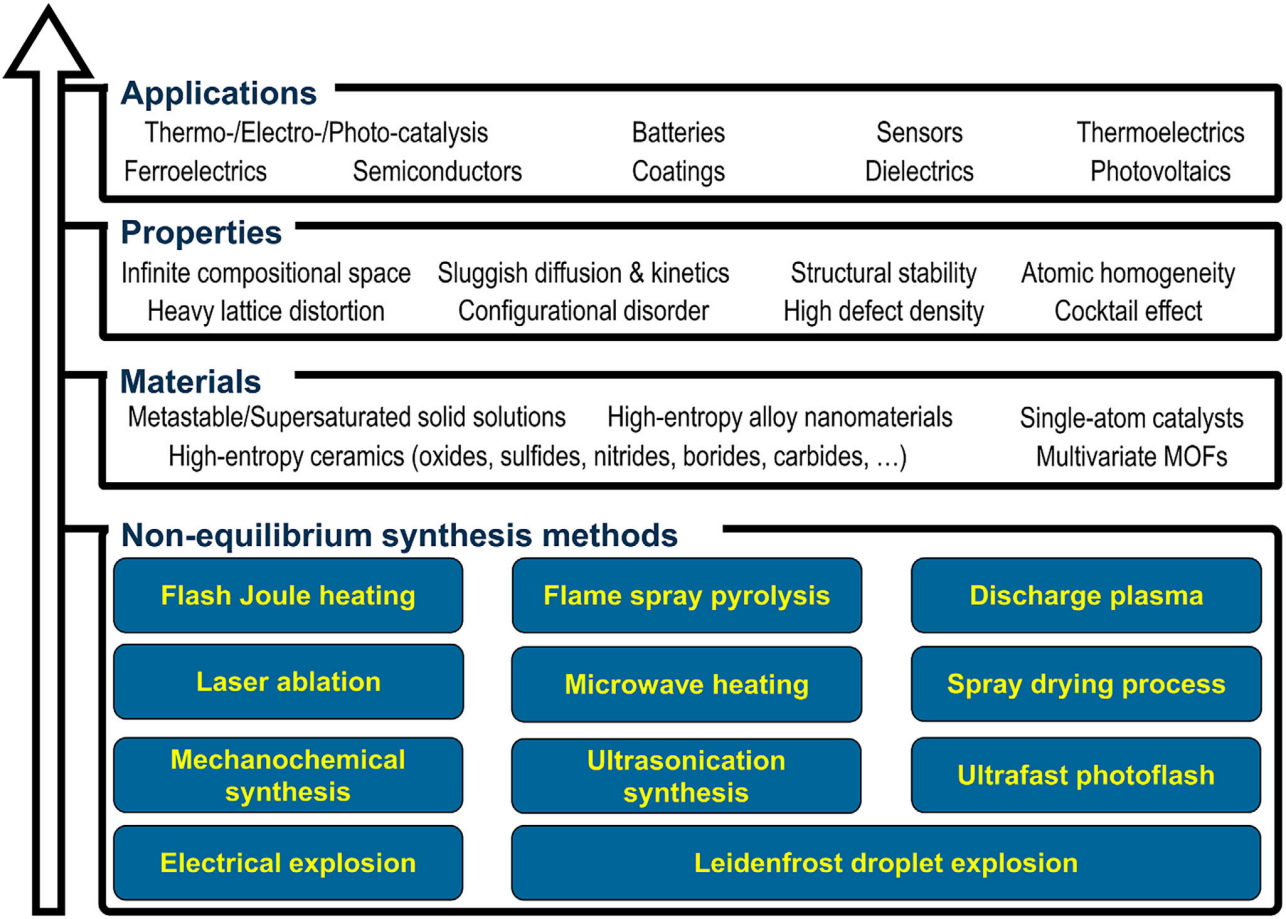
Summary of the synthesis‐material‐property‐application combinations based on non‐equilibrium synthesis.

This review highlights the technical details and transformative potential of non‐equilibrium synthesis methods for creating multicomponent metastable materials (Figure 4). By surpassing the solubility limits dictated by conventional solid‐state thermodynamics, these methods unlock the formation of supersaturated solid solutions, high‐entropy alloys, high‐entropy ceramics, and multivariate MOFs, vastly expanding the compositional landscape and structural diversity of inorganic nanomaterials [20]. Beyond composition, the extreme reaction conditions intrinsic to these methods can impart other structural characteristics, including high defect densities, severe lattice distortion, and small particle sizes. The uniform dispersion of multiple elements within the same crystal lattice at the atomic scale gives rise to unconventional properties, such as enhanced structural stability induced by sluggish kinetics [19]. More significantly, the intricate interplay among diverse elements fosters unprecedented synergies, driving breakthroughs in material performance [54]. These distinctive attributes are particularly sought after in energy, electronic, and functional materials [9, 10, 11, 55, 56], and most prominently in catalysis [8, 37, 57, 58], where unconventional compositions and structures have demonstrated remarkable improvements in activity, stability, and selectivity. With their ability to break traditional limitations and unlock novel functionalities, non‐equilibrium synthesis methods are set to redefine the frontiers of inorganic materials science.

## 3 Non‐Equilibrium Synthesis Methods

### 3.1 Flash Joule Heating

**FIGURE 5:**
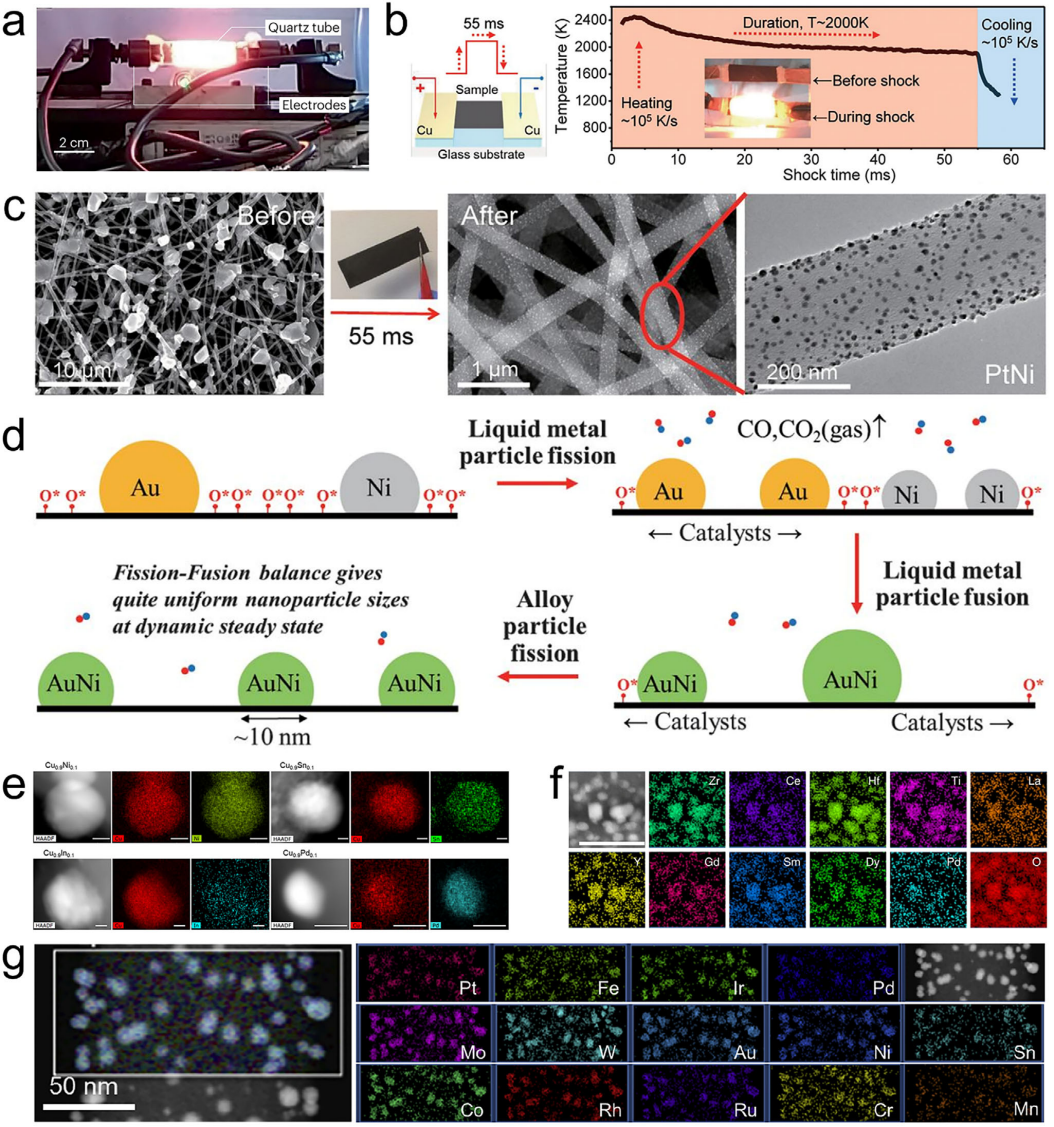
Flash Joule heating. (a) Quartz tube configuration flash Joule heating. Reproduced with permission [59]. Copyright 2025, Springer Nature; (b) Carbon sheet configurational flash Joule heating and the temporal evolution of temperature during the thermal shock, (c) precursor salts before heating and alloy nanoparticles on carbon nanotube after heating, and (d) alloying mechanism in flash Joule heating process. Reproduced with permission [68]. Copyright 2018, AAAS; (e) Metastable Cu‐based alloys. Reproduced with permission [71]. Copyright 2020, AAAS; (f) 10‐element fluorite oxide. Reproduced with permission [72]. Copyright 2021, Springer Nature; and (g) 15‐element alloy nanoparticles. Reproduced with permission [15]. Copyright 2021, Cell Press.

Flash Joule heating [59] (also called carbothermal shock, high‐temperature shock, flash electrothermal process, ultrafast heating, flash sintering, or rapid Joule heating, etc.) (Figure 5a), is the most popular non‐equilibrium synthesis method, with proposed applications in nano‐manufacturing [60], graphene synthesis [61], diamond growth [62], plastic depolymerization [63], thermochemical synthesis [46], sintering of ceramics [64], solid‐waste recycle [65], single‐atom catalysis [44], soil remediation [66], and meat preservation [67]. Typically, flash Joule heating is a direct heating process, where a high‐power, short‐duration electrical pulse is applied directly to a resistive material. This rapidly heats the target material across a broad temperature range, followed by rapid cooling after the pulse. The target material itself can serve as the resistive material or it can be in contact with a separate resistive material. In flash Joule heating reactors, the reactant feedstock is often loaded into a fused quartz vessel, comprising an insulating tube capped at both ends with electrodes made of brass or graphite [61]. Alternatively, the reactants can be sandwiched between two metal electrodes or carbon paper [64]. In generally, flash Joule heating can use any system that applies sufficient voltage across the reactant medium to achieve rapid heating. Initially, capacitor‐based systems were used, where the energy delivered was determined by the capacitors' total capacitance and charging voltage. These systems can achieve high power outputs (up to 1 MW) but are limited to short durations (typically sub‐second) and offer limited control over temperature and heating rates. In contrast, non‐capacitor systems use programmable power supplies that allow precise control of voltage and current for accurate temperature regulation and continuous heating or cooling without the need for recharging. Although these systems have lower power output compared to capacitor‐based systems, they offer superior energy controllability [59].

In the flash Joule heating process, the instantaneous ultrahigh energy input enables heating and cooling rates of up to 10^5^ K/s, reaching temperatures above 3000 K (Figure 5b) [44, 68]. These non‐equilibrium conditions allow precise kinetic control for the structural engineering of metastable materials. For example, metastable phases such as 1T‐MoS_2_ and 1T‐WS_2_ [69], α‐MoC_1‐x_, and η‐MoC_1‐x_ [70] can be selectively synthesized. It also enables the incorporation of immiscible elements into metastable solid solutions. A mixed metal salt precursor supported on a substrate can rapidly transform into well‐dispersed alloy nanoparticles within a few milliseconds of heating (Figure 5c) [68]. During the flash Joule heating process, metal salt precursors rapidly melt and are reduced to form liquid metal droplets. The residual oxygen (O^*^) on the defective carbon support surface, together with the metal catalysts, facilitates carbothermal reduction, releasing CO gas. This reaction drives the active movement and fission of liquid metal droplets under high temperatures, causing them to split into smaller droplets for more efficient harvesting of surface O^*^. Under the intense thermal shock, the liquid metal droplets continuously undergo fission to enhance dispersion. Meanwhile, the high mobility of the droplets enables frequent collisions and fusion, leading to the formation of single‐phase alloy nanoparticles. Throughout the ∼55 ms high‐temperature exposure, these fission and fusion events occur repeatedly, eventually establishing a dynamic steady state. This high‐frequency fission‐fusion dynamic not only ensures uniform nanoparticle sizes (around 10 nm) but also promotes homogeneous alloying of the elements. Higher O^*^ concentrations and metals with greater catalytic activity enhance particle mobility and increase the frequency of fission‐fusion events, improving both dispersity and alloy homogeneity. In contrast, lower O^*^ concentrations restrict particle movement, resulting in slower coarsening. Additionally, the wide solubility range of liquid alloys at temperatures around 2000 K further facilitates the fusion of different droplets into single‐phase alloys. Ultimately, through the catalytically driven fission‐fusion mechanism of liquid metals, the flash Joule heating process enables the rapid and efficient formation of uniformly dispersed alloy nanoparticles (Figure 5d).

To date, the flash Joule heating technique has achieved the versatile synthesis of metastable and high‐entropy solid‐solution nanomaterials. For example, a binary alloy nanoparticle library was established by incorporating various immiscible elements, such as Ni, In, and Sn, into the Cu lattice (Figure 5e) [71]; Driven by the combined effects of ultrafast reaction kinetics and configurational entropy, 15‐element PtPdRhRuIrAuCuFeCoNiCrMnWMoSn high‐entropy alloy nanoparticles were produced (Figure 5g) [15]; A great variety of high‐entropy ceramic nanomaterials, such as 10‐element (ZrCeHfTiLaYGdSmDyPd)O_2_ oxide (Figure 5f) [72], (FeCoNiCuMn)S sulfide [73], and refractory (VNbMoTaW)C carbides [74], have been fabricated by this technology. Furthermore, Flash Joule heating not only enables ultrafast heating and cooling but also allows for controlled heating and cooling rates, as well as adjustable annealing times. This flexibility facilitates kinetically controlled tuning of the fundamental structures of inorganic materials. For example, after the initial ultrafast heating, a rapid re‐heating to 1100 K followed by a 5‐min thermal annealing can induce a disorder‐to‐order transition, enabling the phase transformation from a high‐entropy alloy to a high‐entropy intermetallic [75]; amorphous multi‐component metallic glass nanoparticles can also be directly prepared [76]; Meanwhile, properties such as morphology, defects, and particle size can also be tuned. Owing to their flexible and tunable composition and structure, metastable and high‐entropy nanomaterials synthesized via flash Joule heating exhibit broad potential for applications in catalysis, batteries, and a wide range of functional materials [60, 77]. However, while heating rates can be readily controlled, the achievable cooling rate is often constrained by heat transfer to the surroundings, making rapid quenching more difficult at larger scales. This factor may prove to be a limitation or at least an important design consideration for scale‐up.

Flash Joule heating offers advantages over traditional combustion‐based and wet‐chemistry methods for metastable solid solution design, thanks to its high energy efficiency, ultrafast reaction time, solvent‐free process, minimal heat loss, versatile operation, and compact reactor design. However, challenges remain, including limited temperature control and heat distribution issues in large‐scale production. A deeper understanding of multifield coupling effects is essential for improving controllability, and some studies focused on addressing these challenges. For example, the hydrogen‐substituted graphdiyne‐assisted ultrafast sparking technique utilizes an aerogel substrate with a high surface area and a high volume‐to‐mass ratio, reaching an ultra‐high temperature of 3286 K within just 8 ms, which enables the universal synthesis of high‐entropy alloys, high‐entropy ceramic nanomaterials, and single‐atom catalysts [78]. The recently reported roll‐to‐roll carbothermal shock technology enables one‐step synthesis and continuous manufacturing, achieving a scalable production rate of 7 m/min for high‐entropy alloys, oxides, and multi‐element hetero‐structured catalysts. It also allows for the large‐scale fabrication of electrodes up to 10 × 100 cm^2^, paving the way for high‐throughput synthesis and industrial applications of ultrafast heating technology [79].

### 3.2 Flame Spray Pyrolysis

**FIGURE 6:**
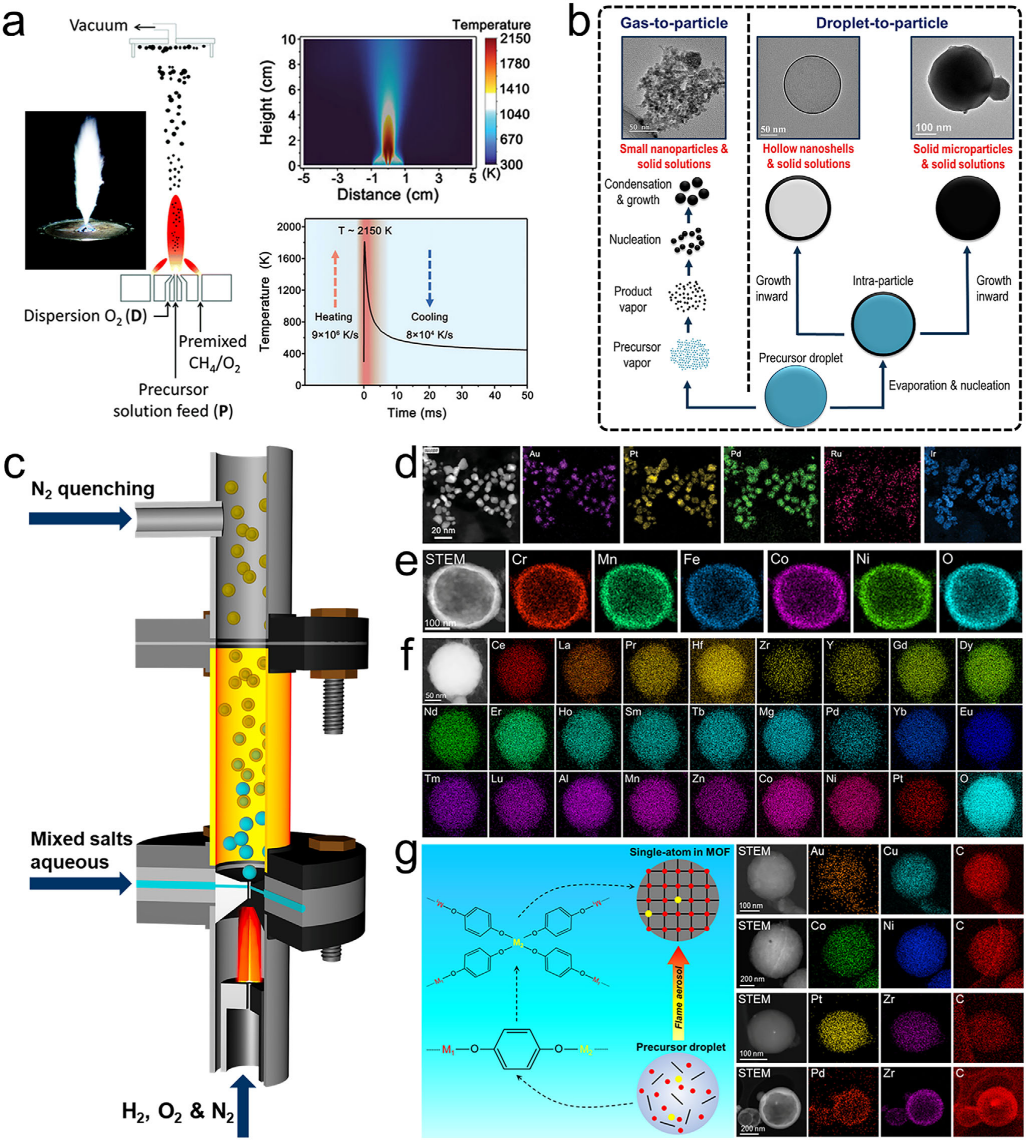
Flame spray pyrolysis. (a) Typical flame spray pyrolysis setup, temperature distribution along the gas flow direction. Reproduced with permission [80]. Copyright 2016, Royal Society of Chemistry; and temperature‐time curve in the core area of the flame. Reproduced with permission [83]. Copyright 2024, Wiley‐VCH. (b) Particle formation mechanisms in flame spray pyrolysis. (c) Schematic of a modified flame reactor. Reproduced with permission [16]. Copyright 2024, Cell Press; (d) AuPtPdRuIr high‐entropy alloy nanoparticles formed by a gas‐to‐particle route. Reproduced with permission [83]. Copyright 2024, Wiley‐VCH. (e) (CrMnFeCoNi)_3_O_4_ high‐entropy spinel oxide formed in a droplet‐to‐particle route. Reproduced with permission [16]. Copyright 2024, Cell Press; (f) a 25‐element (CeLaPrHfZrYGdDyNdErHoSmTbMgPdYbEuTmLuAlMnZnCoNiPt)O_2_ high‐entropy fluorite oxide fabricated using the modified flame reactor of panel (c). Reproduced with permission [99]. Copyright 2025, American Chemical Society; (g) Schematic of the multivariate MOF solid solution formation in the modified flame reactor and the produced bi‐metallic MOFs. Reproduced with permission [98]. Copyright 2024, Springer Nature.

Flame spray pyrolysis (also called flame aerosol synthesis), is the oldest and the most widely used nanomaterial synthesis method in industry, known for its high yield and scalability [42, 80]. This technology enables continuous, one‐step production, supporting the industrial‐scale manufacturing of inorganic nanomaterials. Companies like Cabot, Tronox, and Evonik use flame spray pyrolysis to produce millions of tons of carbon black, fumed silica, and ceramic oxides (TiO_2_, Al_2_O_3_, ZrO_2_) annually, with individual reactors achieving outputs of several tons per hour [81, 82]. Typically, in flame synthesis, a precursor solution or vaporized gaseous precursor is injected into a flame. The precursor can be an organic solution that sustains the flame through its combustion enthalpy or an aqueous solution that relies on an external flame for energy. In the latter case, it is often called flame‐assisted spray pyrolysis. At high temperatures, the precursor undergoes rapid thermal decomposition, leading to rapid nucleation, growth, and eventual formation of nanoparticles in the gas phase or within droplets (Figure 6a) [80]. During synthesis, key nanoparticle properties, such as size, morphology, surface area, and crystal structure can be flexible tailored by adjusting reaction parameters like gas and precursor flow rates, reaction temperature, and reactor configuration [80, 81, 82]. More importantly, it provides ultrafast heating (9 × 10^6^ K/s) and cooling rates (8 × 10^4^ K/s), up to 2000 K reaction temperature, and short residence time (∼5 ms) [83], creating favorable kinetic conditions for integrating immiscible elements and synthesizing metastable and high‐entropy solid solutions [84].

In general, the particle formation in flame spray pyrolysis process involves two mechanisms: gas‐to‐particle and/or droplet‐to‐particle paths (Figure 6b) [80, 81, 82]. The gas‐to‐particle pathway typically occurs when the precursor itself is a vapor or it is dissolved in a combustible organic solvent, and the reaction temperature is high. In this process, precursor microdroplets are pyrolyzed into a supersaturated vapor, where product particles nucleate and grow directly from the gas phase. The droplet‐to‐particle pathway typically occurs when the precursor is an aqueous solution, the reaction temperature is relatively low, and particle formation is driven by rapid droplet evaporation. In this process, evaporation increases the solute concentration at the droplet surface. Because the evaporation rate exceeds the rate of solute diffusion within the droplet, the surface reaches supersaturation first, leading to nucleation of a solid phase at the outer surfaces of droplets and subsequent inward growth. This often results in the formation of hollow nanoshells [85, 86]. Depending on precursor concentration and other reaction conditions, solid microparticles can also be formed.

Both formation pathways align with the principles of non‐equilibrium synthesis, enabling the production of metastable and high‐entropy materials. For example, AuPtPdRuIr high‐entropy alloy nanoparticles (Figure 6d) were formed by a gas‐to‐particle route [83] and (CrMnFeCoNi)_3_O_4_ high‐entropy spinel oxide nanoshells (Figure 6e) were produced by a droplet‐to‐particle route [83]. This approach also enables deposition of smaller multi‐component or high‐entropy nanoparticles on various support materials [87, 88]. Furthermore, tunable composition and structure, along with the ability to incorporate immiscible elements into various crystal lattices, make flame spray pyrolysis a highly versatile technique for developing inorganic functional and energy materials with promising applications in catalysis [81], sensors [89], and Li‐ion batteries [90]. For example, the metastable CoCu_2_O_3_ nanocrystal exhibits p‐type semiconducting behavior with a narrower bandgap than CuO or CO_3_O_4_, demonstrating promising performance for ultra‐sensitive molecular sensing at sub‐ppb levels [91], while doping Pd into the lattice of n‐type In_2_O_3_ enabled a highly responsive H_2_ sensor [92]. Similarly, in the field of catalysis, flame spray pyrolysis enables the direct doping of atomically dispersed active sites into the support lattice, allowing the formation of single‐atom catalysts, such as the Pt‐ZrO_2_ single‐atom catalysts that have demonstrated excellent performance in CO oxidation, methane combustion, and methane reforming reactions [93]. Due to the intense flame reaction and the differences in atomic radii and electronegativity among elements in solid solutions, the produced materials often exhibit high defect densities and heavy lattice distortions, which can be beneficial for catalysis. For instance, lattice strain in (FeCoNiMnCr)_3_O_4_ high‐entropy spinel oxides can be tuned via quenching to enhance their OER electrocatalytic performance [94].

The conventional flame spray pyrolysis involving high reaction temperatures typical of flames (∼2000°C) limits its ability to synthesize thermally sensitive materials like MOFs. Additionally, the use of organometallic precursors increases costs and poses environmental concerns. A modified flame aerosol reactor has addressed these challenges (Figure 6c) [95]. It uses a H_2_‐rich flame to provide the reaction energy and employs aqueous precursors. A converging‐diverging nozzle separates the flame from the material formation zone, allowing precise control over the reaction temperature. The redox environment within the reaction chamber can be tuned by adjusting the H_2_/O_2_ flow rates, enabling in situ reduction for the synthesis of metal nanoparticles [96]. Downstream, rapid quenching with high‐speed cold N_2_ helps maintain metastable phases formed at high temperatures. Combining these advantages, this modified flame reactor significantly expands the range of materials that can be synthesized in flame spray pyrolysis technology. For example, for the first time, mesoporous silica [97] and MOF [98] materials were fabricated by a flame‐driven aerosol process. The modified flame reactor can also overcome thermodynamic immiscibility between elements, enabling the integration of nearly any pair of elements from the periodic table into a single‐phase metastable oxide solid solution with high supersaturation [28], and enabling the general synthesis of high‐entropy nano‐ceramics with flexible composition and structure, including a 25‐component high‐entropy fluorite oxide (Figure 6f) [99]. Furthermore, in its droplet‐to‐particle formation process, two different metal ions can rapidly coordinate with the same organic ligand, forming a single‐phase MOF solid solution with atomically dispersed dopant elements (Figure 6g) [98]. This approach enables the integration of immiscible elements into the MOF lattice, offering a general strategy for the synthesis of multivariate MOFs, such as Au‐Cu HKUST‐1, Co‐Ni PBA (Prussian blue analogue), Pt‐Zr UiO‐66‐NH_2_, and Pt‐Zr FMA (fumaric acid) (Figure 6g). Materials synthesized using this advanced flame aerosol technique, including metal nanoparticles, metastable ceramic solid solutions, high‐entropy ceramics, and multivariate MOFs, have demonstrated excellent performance in applications such as H_2_ sensing [100], dry reforming of methane [28, 97, 101], CO_2_ reduction to CO [16], and CO oxidation [98].

Similar to flame spray pyrolysis, combustion synthesis [102] (also known as self‐propagating high‐temperature synthesis) is a flame‐based non‐equilibrium strategy for preparing nanomaterials. It relies on highly exothermic combustion reactions between oxidizers and fuels such as urea, glycine, or citric acid. Once ignited, the reaction propagates rapidly, reaching temperatures above 1500 K and releasing large amounts of gas. These extreme yet short‐lived conditions drive instantaneous nucleation while suppressing particle growth, resulting in products with high defect densities, metastable phases, and extended solid solubility. The final size, morphology, and phase composition can be tuned by varying the fuel type, fuel‐to‐oxidizer ratio, or solution pH. For example, combustion synthesis has produced MnFeCoNiCu high‐entropy alloys and their spinel oxides under different conditions [103], rock‐salt (CoCuMgNiZn)O high‐entropy oxides [104], and nanocrystalline high‐entropy alloys via sol–gel auto‐combustion [105]. A further advance was achieved using a candle flame method, where metal precursors dissolved in paraffin wax are continuously transported into the flame [106]. At ∼1800 K in the candle flame, reactive intermediate forms and polycyclic aromatic hydrocarbons aggregate into soot, which simultaneously serves as a reductant and support for nucleating metals. Metal ions are dynamically trapped within the soot, enabling rapid alloying and the formation of high‐entropy nanomaterials within milliseconds. Molecular dynamics simulations show that the high flame temperature promotes PAH fragmentation into reactive carbon species that interact strongly with metals, stabilizing interfaces and facilitating the co‐clustering of immiscible elements. This process allows precise control of product size from single atoms to nanoclusters to nanoparticles simply by adjusting precursor concentration, and up to 25 metals have been uniformly incorporated into a single nanoparticle. Beyond metals, heteroatoms such as P, S, and F can be introduced for further functionality. Importantly, scaling by enlarging wicks or using multiple candles enables gram‐scale production (∼1 g/h) per candle.

### 3.3 Discharge Plasma

**FIGURE 7:**
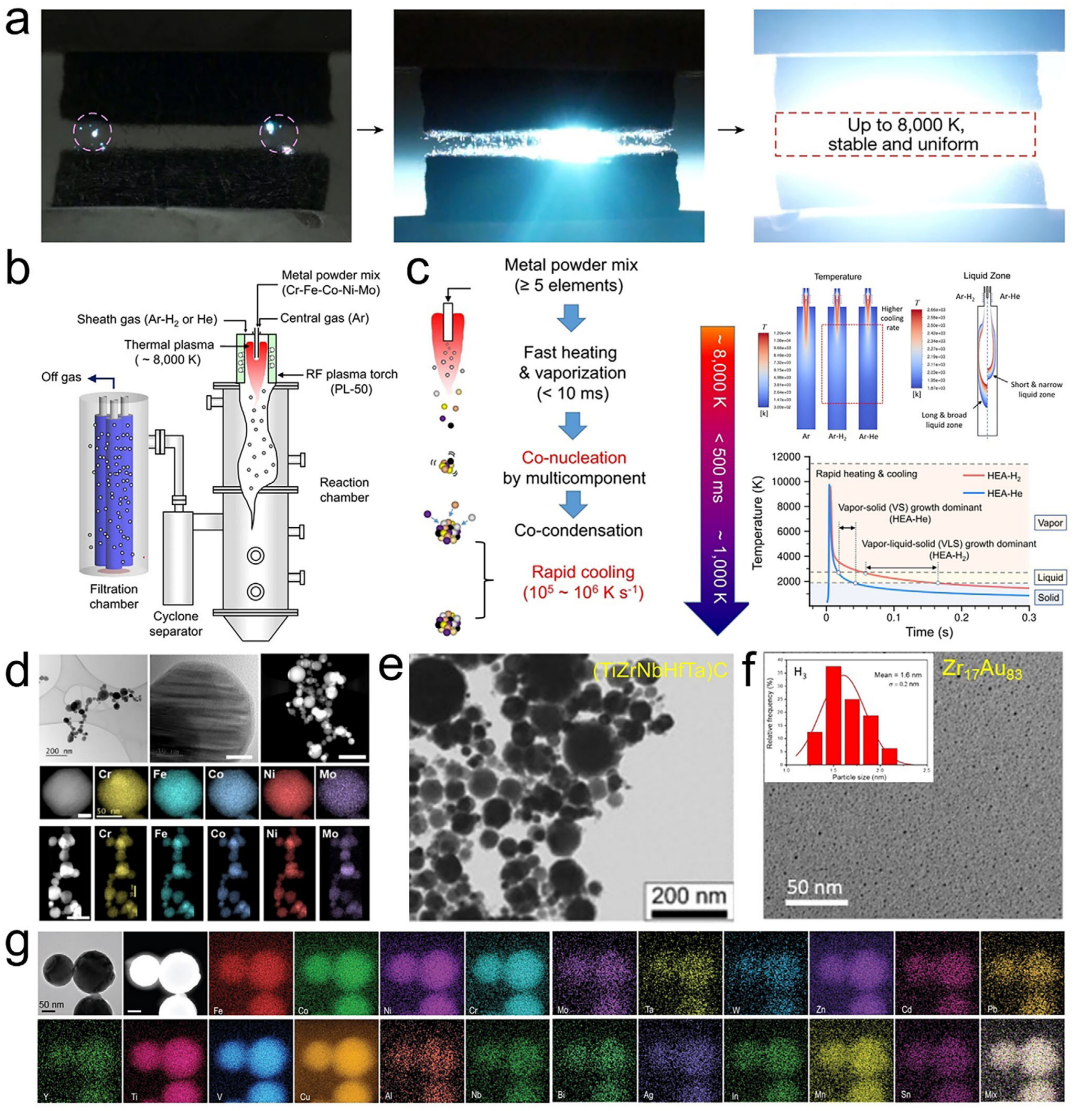
Discharge plasma. (a) Photographs of the volumetric plasma generation using carbon‐fiber‐tip electrodes. Reproduced with permission [108]. Copyright 2023, Springer Nature; (b) Typical RF thermal plasma synthesis setup, (c) schematic of the nanoparticle formation mechanism in a plasma synthesis process, the temperature and the liquid zone distribution along the plasma direction, and the heating/cooling curve in the reactor, and (d) the produced CrFeCoNiMo high‐entropy alloy. Reproduced with permission [113]. Copyright 2024, Springer Nature; (e) (TiZrNbHfTa)C high‐entropy spinel carbide. Reproduced with permission [114]. Copyright 2025, Elsevier; (f) Ultrasmall Zr_17_Au_83_ alloy. Reproduced with permission [118]. Copyright 2020, Cell Press; (g) 21‐component FeCoNiCrYTiVCuAlNbMoTaWZnCdPbBiAgInMnSn high‐entropy alloy nanoparticles. Reproduced with permission [119]. Copyright 2022, Oxford University Press.

Discharge plasma is the highest‐temperature and most extreme non‐equilibrium synthesis method, with temperatures that can exceed 10 000 K. It represents a broad class of techniques that use electrically induced plasma to generate highly reactive environments for material synthesis. These methods are known by various names in the literature, such as spark plasma, arc plasma, thermal plasma, and oscillatory spark discharge, depending on their specific plasma generation mode, energy input, and operational configuration. Despite these variations, they share a common mechanism: electrical breakdown of gases leading to plasma formation, which enables the rapid conversion of precursors into solid materials under extreme and far‐from‐equilibrium conditions [107]. For example, plasma generated between a pair of carbon fiber tip electrodes can provide a stable high temperature of 8000 K at atmospheric pressure (Figure 7a), enabling the synthesis of ultra‐high‐temperature ceramics, refractory alloys, and carbon nanotubes in seconds [108]. In addition, based on differences in ionization degree and thermalization of electrons (i.e., whether electrons in the plasma are at the same effective temperature as neutral molecules and ions), plasmas can be categorized into thermal plasmas and non‐thermal plasmas. Thermal plasmas typically take the form of arc discharges and inductively coupled plasmas (ICP) and operate near atmospheric pressure. In these plasmas, energy input and collision rates among ions, neutral atoms, and electrons are sufficiently high to establish the same high temperature for electrons, ions, and atoms, often in the range of 5000 to over 10 000 K. This results in a high degree of ionization, i.e., a large fraction of the gas is ionized. Because all species are at similarly high temperatures, thermal plasmas drive intense chemical reactivity and high energy density for metastable nanomaterial production. In contrast, a non‐thermal plasma is typically generated through low‐power or pulsed discharges at reduced pressure, such as low‐pressure radio‐frequency (RF) plasma, dielectric barrier discharges (DBD), and glow discharges. These plasmas rely on electric fields to selectively accelerate electrons, while the bulk gas remains only mildly heated due to the low rate of electron‐ion collisions. As a result, electrons in non‐thermal plasma can reach temperatures of several thousand Kelvin (1–10 eV), while ions and neutral gas molecules remain near ambient temperature. Although the neutral gas temperature is low, the presence of high‐energy electrons initiates chemical reactions, dissociation, and radical formation [109]. This enables high‐entropy materials synthesis and surface modification under relatively mild conditions. For example, the MoNbTaVWB_10_ high‐entropy boride [110] and PtCrTaVFeAl high‐entropy alloy nanoparticles with great methanol oxidation electrocatalytic performance [111] were synthesized using a non‐thermal plasma. Moreover, non‐thermal plasma post‐treatment was shown to enrich oxygen vacancies in a (NiCoCrMnMo)_3_O_4_ high‐entropy oxide prepared by thermal plasma, improving its activity as a water‐splitting catalyst [112].

The extremely high reaction temperature and rapid heating/cooling rates (10^5^–10^6^ K/s) in thermal plasma provide ideal conditions for non‐equilibrium synthesis (Figure 7b,c). The high temperature, often exceeding 8000 K, is sufficient to instantly vaporize solid raw materials, thereby allowing the use of not only liquid‐ and gas‐phase precursors but also solid‐phase feedstocks. The material formation mechanism typically follows a gas‐to‐particle route, involving vaporization of the feedstock, nucleation, and subsequent condensation into nanoparticles (Figure 7c) [113]. In a typical synthesis, the precursor, such as a mixture of metal powders, is continuously fed into a high‐temperature plasma jet. Within milliseconds, the injected feedstock is evaporated in the plasma core, generating elemental metal vapors. As the plasma jet expands, these vapors mix and form a homogeneous multicomponent vapor within the reactor zone. Upon exiting the high‐temperature region, the supersaturated vapor experiences rapid quenching, leading to the nucleation of nanoparticles, followed by particle growth through the co‐condensation of metal monomers, forming homogeneous alloy nanoparticles (Figure 7d). The growth pathway of nanoparticles is governed by multiple factors, such as discharge power, reaction temperature, gas flow rate and composition, and reactor geometry. Among these, this study shows that the plasma gas composition plays a critical role in shaping the thermal history during particle flight [113]. In hydrogen‐containing plasma, the extended residence time and broader liquid‐phase region can promote a Vapor–Liquid–Solid growth mechanism, where the supersaturated vapor tends to first condense into a liquid phase, and nanoparticles form and grow within the molten state. In contrast, helium plasma leads to much faster cooling, resulting in a narrow liquid zone and favoring a Vapor–Solid mechanism, in which nanoparticles nucleate and grow directly from the gas phase (Figure 7c).

Discharge plasmas provide several key advantages for materials synthesis. The extremely high temperature enables the vaporization of a wide range of precursors, including refractory metals and ceramics, making them suitable for synthesizing compositionally complex and high‐melting‐point materials, such as the (TiZrNbHfTa)C high‐entropy carbide (Figure 7e) [114]. The rapid heating and quenching rates facilitate production of metastable phases with immiscible metals [115, 116, 117] and fine nanoparticles. For example, oscillatory spark discharge occurring within a few microseconds can directly vaporize metal electrodes and cool them at an extremely high rate (10^7^–10^9^ K/s), enabling the general synthesis of 55 different ultra‐small (1–5 nm) alloy nanoparticles composed of 2–6 elements, including immiscible and high‐melting‐point combinations such as WAu and ZrAu (Figure 7f), which also demonstrated excellent catalytic performance in methanol oxidation reaction (MOR), ethanol oxidation reaction (EOR), and formic acid oxidation (FAOR) reactions [118]. Additionally, the high energy density and reactive environment promote efficient mixing of multiple elements, supporting the formation of homogeneous 21‐component FeCoNiCrYTiVCuAlNbMoTaWZnCdPbBiAgInMnSn high‐entropy alloy nanoparticles (Figure 7g) [119]. Discharge plasma fabricated metastable and high‐entropy nanomaterials have been produced and tested in many other applications, such as the (NiFeMnCuZn)_3_O_4_ oxide for OER electrocatalysis [120], (MnFeCoNiCu)_3_O_4_ oxide for supercapacitor [121], and (MoWVNbTa)C carbide for HER electrocatalysis [122].

### 3.4 Laser Ablation

Laser ablation is the fastest non‐equilibrium synthesis method. Due to the ultra‐short pulse duration, the laser peak intensity can exceed 10^12^ W/cm^2^ at the focal point, enabling extreme energy deposition within femtoseconds [123]. The synthesis process involves the irradiation of precursors with high‐energy laser pulses to induce rapid heating, decomposition, and plasma formation, followed by ultrafast cooling to yield nanomaterials, which enables the generation of nanomaterials with precisely tailored morphology, composition, and particle size from quantum dots to sub‐micrometer spheres [124]. Meanwhile, laser ablation has the unique capability unattainable by any other method to digitally and selectively synthesize, modify, and integrate nanomaterials with high spatial precision, enabling mask‐free, programmable fabrication even on complex 3D structures. By serving as a localized heat source, laser enables selective photothermal or photo‐thermo‐chemical reactions at arbitrary positions, while also leveraging optical forces, photochemical interactions, and light–matter coupling to achieve advanced processing techniques such as nano‐welding of percolation networks and laser thinning of 2D materials [125].

**FIGURE 8:**
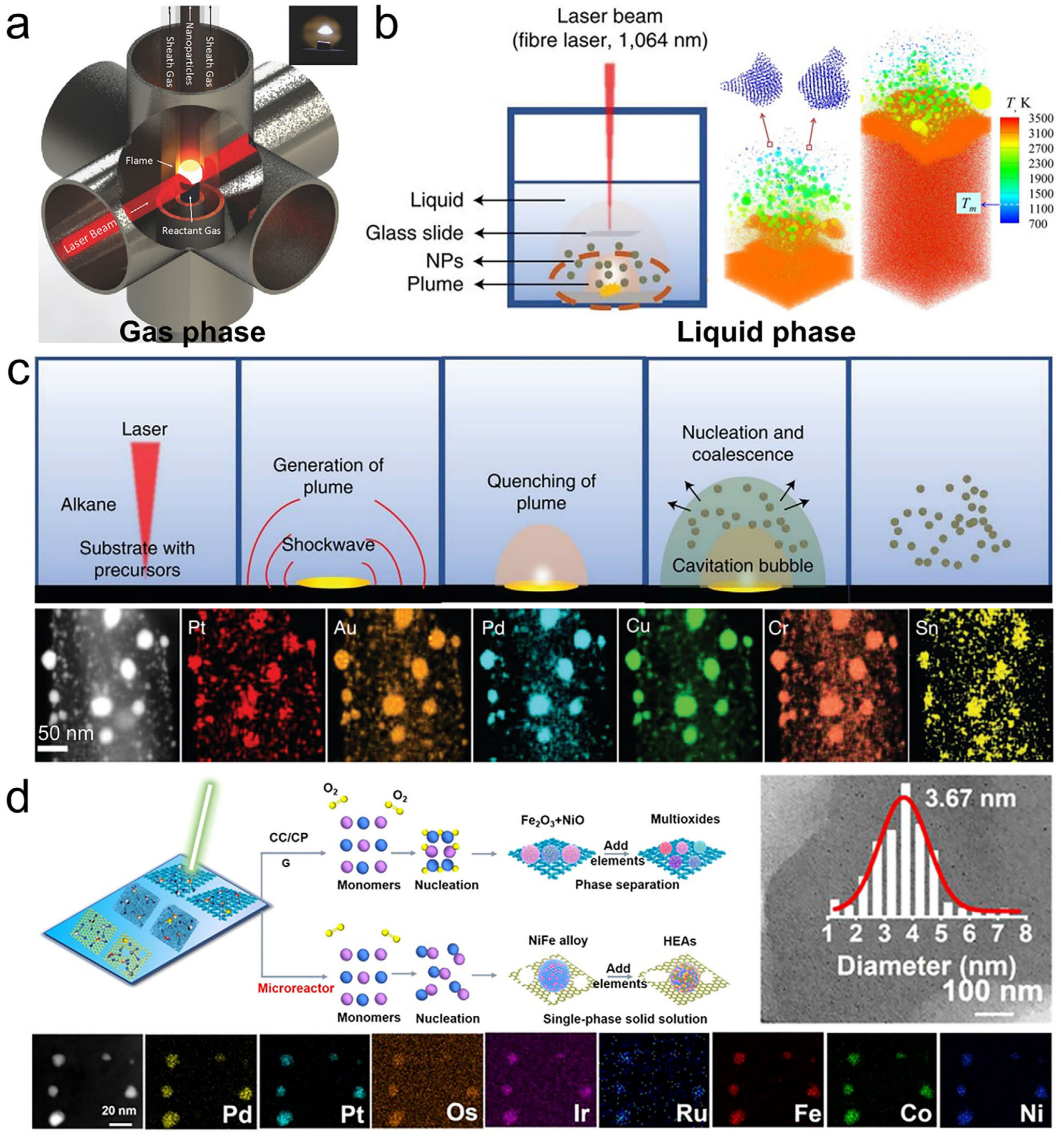
Laser ablation. (a) Schematic of a gas‐phase laser pyrolysis reactor. Reproduced with permission [126]. Copyright 2016, Wiley‐VCH; (b) Schematic of liquid‐phase laser reaction, and the simulated temperature profile of the Ag film target under laser ablation in liquid at 3 ns after irradiation, at absorbed laser fluences of 400 (left) and 700 (right) J/m^2^. Reproduced with permission [135]. Copyright 2017, Elsevier; (c) High‐entropy alloy nanoparticle formation mechanism via liquid‐phase laser ablation and the resulting PtAuPdCuCrSn on a carbon nanotube. Reproduced with permission [136]. Copyright 2022, Springer Nature; (d) High‐entropy alloy nanoparticle formation mechanism by direct laser ablation of metal mixed‐salt precursors supported on graphene in air, the corresponding particle size distribution at a precursor concentration of 0.01 m, and the resulting octonary PdPtOsIrRuFeCoNi nanoparticles. Reproduced with permission [141]. Copyright 2024, American Chemical Society.

Depending on the reaction environment, laser synthesis can be broadly categorized into gas‐phase (laser pyrolysis) and liquid‐phase processes. The gas‐phase laser synthesis (Figure 8a) [126] is similar to the gas‐to‐particle process in flame spray pyrolysis and discharge plasma, where reactive gas mixtures or vaporized precursors, such as metal–organic compounds or volatile halides, are exposed to a focused laser beam, typically in a continuous or pulsed mode. The intense laser energy induces rapid thermal decomposition of the precursors, driving nucleation and growth of nanoparticles directly in the gas phase. The process is typically conducted in a flow‐through reactor equipped with inert or reactive carrier gases and downstream collection units such as filters or impactors [127, 128, 129]. Owing to the extremely high local temperatures and fast quenching rates, laser pyrolysis enables the mixing of immiscible elements into metastable nanostructures, such as boron hyper‐doped silicon nanoparticles [130], SiGe alloy nanoparticles [131] and doping Au into TiO_2_ for photocatalytic water splitting [132].

Liquid‐phase laser synthesis (Figure 8b) typically involves focusing a pulsed laser onto a solid target or a precursor solution submerged in a liquid medium. Upon laser irradiation, a transient high‐temperature plasma plume is generated at the solid–liquid interface or within the liquid bulk, which rapidly cools to yield nanoparticles. This approach enables the surfactant‐free synthesis of a wide variety of nanomaterials with clean surfaces and controlled size distributions. By tuning the laser fluence, pulse duration, and solvent properties, the laser ablation and fragmentation of solids, or photothermal reduction of dissolved precursors, can be realized [133, 134]. More importantly, atomistic simulations reveal that in liquid‐phase laser ablation of thin Ag films, the extreme heating rate can reach ∼10^14^ K/s during femtosecond laser excitation, while the cooling rate of the ablation plume in the metal–water mixing region exceeds 10^12^ K/s (Figure 8b) [135], enabling ultrafast nucleation and solidification of nanoparticles under extreme non‐equilibrium conditions, making it particularly effective for producing metastable phases, defect‐rich structures, and high‐entropy nanoparticles.

For example, the liquid‐phase laser scanning ablation has been developed as a general method to fabricate high‐entropy alloy and ceramic nanoparticles in nanoseconds [136]. In the typical synthesis, metal chlorides of equal molar ratio were first loaded onto a substrate, such as carbon nanofibers. Then, the substrate was transferred to hexane and irradiated by laser pulses at room temperature with a constant average power density of 2 × 10^5^ W/cm^2^ and a peak pulse power density of 2 × 10^9^ W/cm^2^. The formation of high‐entropy nanoparticles follows a photothermal evaporation mechanism (Figure 8c). Upon irradiation, the laser pulse penetrates the transparent liquid medium and interacts with the substrate coated with multicomponent precursors. The intense optical field and rapid localized heating induce precursor melting or decomposition, resulting in the formation of a high‐temperature, high‐pressure plume composed of atoms, ions, clusters, and vapor. This transient homogeneous multi‐element mixture promotes the formation of solid‐solution phases. Recoil from the expanding plume generates shockwaves and cavitation bubbles, which further facilitate plume dispersion and energy release. As the plume and bubbles rapidly cool through ultrafast heat exchange with the surrounding liquid, nuclei of solid solutions form and coalesce into nanoparticles. The extreme quenching rates suppress phase separation, enabling the formation of high‐entropy alloys (Figure 8c) and various high‐entropy ceramics, including oxide, sulfide, boride, phosphide, and nitride. For example, PtIrCuNiCr and PtAuPdFeNi produced by this route showed excellent performance as HER and OER electrocatalysts. In addition, support‐free high‐entropy nanomaterials can also be fabricated by laser irradiation of solid targets immersed in liquid, such as RE_2_(Ce_0.2_Zr_0.2_Hf_0.2_Sn_0.2_Ti_0.2_)_2_O_7_ (RE = Sm, Eu, Er) oxides [137], AlCrCuFeNi alloy nanocolloids [138], and refractory HfNbTaTiZr for photothermal therapy [139].

Additionally, in air, high‐entropy alloy and ceramic nanoparticles can also be synthesized by direct laser irradiation of mixed precursor salts on a substrate [140]. The substrate choice is crucial in this process. For example, under the same synthesis conditions, mixed metal salts supported on carbon cloth, carbon paper, or commercial graphene tended to form phase‐separated oxides, whereas those on porous graphene yielded single‐phase metastable and high‐entropy alloy nanoparticles (Figure 8d) [141]. The 3D porous graphene substrate acts as a microreactor during the rapid heating and cooling process, effectively suppressing oxygen–metal interactions to promote the formation of pure alloy phases. In addition, its confined microstructure provides favorable vapor pressure conditions, enabling precise control over both particle size (as small as 3.67 nm) and composition. Meanwhile, the air‐based laser ablation provides a convenient and high‐throughput route for accelerating material screening and optimization. By combining active learning with laser scribing, this approach enabled the rapid discovery of quinary PtPdFeCoNi HEA nanoparticles with precisely tuned compositions for high‐performance Li–CO_2_ battery cathodes.

### 3.5 Microwave Heating

**FIGURE 9:**
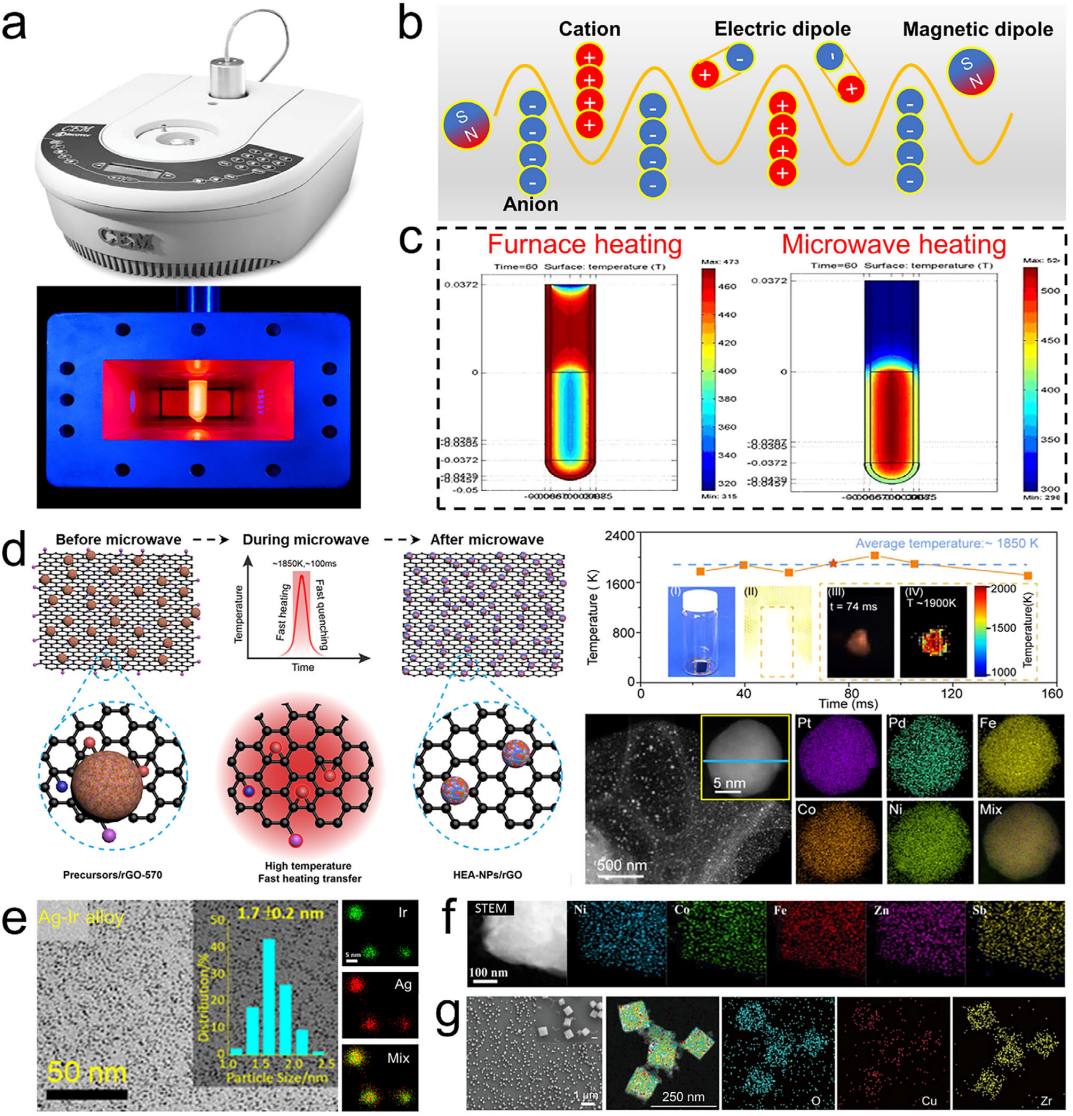
Microwave heating. (a) The microwave reactor and its internal heating environment. Reproduced with permission [142]. Copyright 2014, American Chemical Society; (b) Schematic of dielectric loss, magnetic loss, and conduction loss heating mechanism; (c) Temperature profile of conventional furnace heating and microwave heating. Reproduced with permission [147]. Copyright 2003, Springer Nature; (d) Schematic of high‐entropy alloy nanoparticles formation on graphene oxide, the temperature profile during microwave heating, and the produced PtPdFeCoNi/graphene oxide. Reproduced with permission [148]. Copyright 2021, American Chemical Society; (e) Ultra‐small metastable AgIr alloy nanoparticles. Reproduced with permission [152]. Copyright 2018, American Chemical Society; (f) (NiCoFeZn)Sb high‐entropy antimonide. Reproduced with permission [154]. Copyright 2023, Elsevier; (g) Cu‐Zr bi‐metallic MOF. Reproduced with permission [155]. Copyright 2023, Elsevier.

Microwave heating synthesis (Figure 9a) is a versatile method for the rapid preparation of a wide range of inorganic nanomaterials, including metals, ceramics (oxides, nitrides, carbides, chalcogenides), carbon materials, and complex heterostructures [142]. This approach has also been successfully applied to design diverse morphologies such as nanorods, 2D nanoplates, hollow spheres, and porous frameworks [143, 144, 145]. A key advantage of microwave synthesis lies in its ability to drastically reduce reaction times, from hours to minutes or even seconds, while offering high yields, improved crystallinity, and fine control over size and morphology. Moreover, microwave heating systems can be readily adapted to both solid‐state and solution‐phase reactions, including batch, flow, and hybrid modes, enabling compatibility with both academic laboratories and scalable industrial processes [146].

Microwave synthesis involves the use of microwave‐frequency electromagnetic radiation (typically 2.45 GHz) to heat precursors. At the atomic and molecular level, microwave heating occurs via dielectric loss, magnetic loss, and conduction loss (Figure 9b). Dielectric loss arises from the oscillation of electric dipoles (e.g., polar molecules, bound water, or ionic groups) in response to the alternating electromagnetic field. As these dipoles attempt to realign with the oscillating field, frictional losses convert electromagnetic energy into heat; Magnetic loss involves the reorientation of magnetic dipoles (in materials such as Fe_3_O_4_) under the influence of the alternating magnetic field component of the microwave. This leads to energy dissipation through mechanisms such as magnetic hysteresis, eddy currents, and domain wall motion; Conduction loss occurs in materials with mobile charge carriers (e.g., electrons or ions). In this case, the alternating electric field induces currents that experience resistive (Joule) heating within the material. This mechanism dominates in materials with significant electrical conductivity, such as carbon, graphite, and some semiconductors. These mechanisms can act independently or simultaneously, depending on the dielectric, magnetic, and conductive properties of the materials involved.

Therefore, unlike conventional furnaces that heat materials via surface‐to‐core thermal conduction, microwave heating has unique thermal behavior. First, it allows microwave energy to couple directly with the reaction medium, transferring energy to the material itself rather than the surrounding furnace or container. This results in an inverted temperature profile compared to conventional furnaces, where the material's interior is often hotter than the surface (Figure 9c) [147]. Second, microwave irradiation offers volumetric heating, meaning that in ideal systems, heat is deposited uniformly throughout the sample volume rather than propagating from the surface inward. This reduces the reliance on slow thermal conduction and improves heating efficiency even in relatively large samples [146]. Third, instantaneous heating is a key advantage where microwave energy is absorbed and converted to heat almost immediately, leading to extremely rapid temperature rises. Finally, selective heating arises from the varying ability of different materials to absorb microwave energy, governed by the loss tangent (tan δ) [143]. Some precursors absorb microwaves more efficiently, whereas others absorb weakly. This selectivity allows specific components in a reaction mixture to reach high local temperatures while others remain cooler, potentially offering precise spatial and thermal control.

These distinct features, particularly ultrafast, localized, and selective energy delivery, make microwave heating well‐suited for the non‐equilibrium synthesis of metastable phases and high‐entropy nanomaterials. For example, microwave heating of mixed metal salt precursors supported on graphene oxide (GO) in Ar can produce ∼12 nm PtPdFeCoNi high‐entropy alloy nanoparticles (Figure 9d) [148]. During microwave heating, the GO film effectively absorbs microwave energy through the dipolar polarization of uniformly distributed residual functional group defects. Owing to the intrinsically high thermal conductivity of GO, the localized heat generated at these defect sites is rapidly conducted across the entire film, resulting in an ultrafast temperature rise to approximately 1850 K. This enables uniform heating of the metal salt precursors loaded on the GO surface, leading to their decomposition into liquid metal droplets, which simultaneously reduce the GO to a highly reduced form. As the reduction consumes most of the polar defect groups, the GO transitions from a microwave absorber to a reflector, thereby self‐limiting the heating process. The subsequent rapid quenching of the system, with cooling rates exceeding 6 × 10^4^ K/s, directly solidifies the liquid metals into high‐entropy alloy nanoparticles, effectively preventing elemental segregation and phase separation. This method can be extended to 1D carbon nanotubes and 3D carbonized wood substrates, and is compatible with roll‐to‐roll processing, providing a feasible route for scalable manufacturing of high‐entropy nanomaterials. In addition, the microwave heating generated high‐entropy alloy nanoparticles demonstrated good performance for ORR and OER electrocatalysis [149, 150], and the (MgCuNiCoZn)O high‐entropy oxide worked well as a Li‐ion battery anode material [151].

Liquid‐phase microwave heating enables precise control over particle size, morphology, and structure within metastable phases. For example, immiscible Ag–Ir alloy nanoparticles as small as 1.7 nm (Figure 9e) [152], as well as 2–6 nm immiscible Rh–Ag and Rh–Au alloy nanoparticles [153], can be synthesized using this method, and exhibit excellent catalytic performance in alkene hydrogenation reactions. The NiCoFeZnSb high‐entropy antimonides (Figure 9f) can be produced as materials for ultra‐high energy density supercapacitors [154]. More importantly, this relatively mild liquid‐phase non‐equilibrium reaction creates favorable conditions for the synthesis of MOFs, enabling the incorporation of metal cations with different valences. In this case, divalent Cu ions were incorporated into Zr‐based MOFs (Figure 9g), which typically feature tetravalent Zr nodes. This combination significantly enhances the material's microwave‐sensitizing effect, modulates ferroptosis‐mediated tumor cell death, and shows great potential for synergistic liver cancer therapy [155].

### 3.6 Spray Drying Process

**FIGURE 10:**
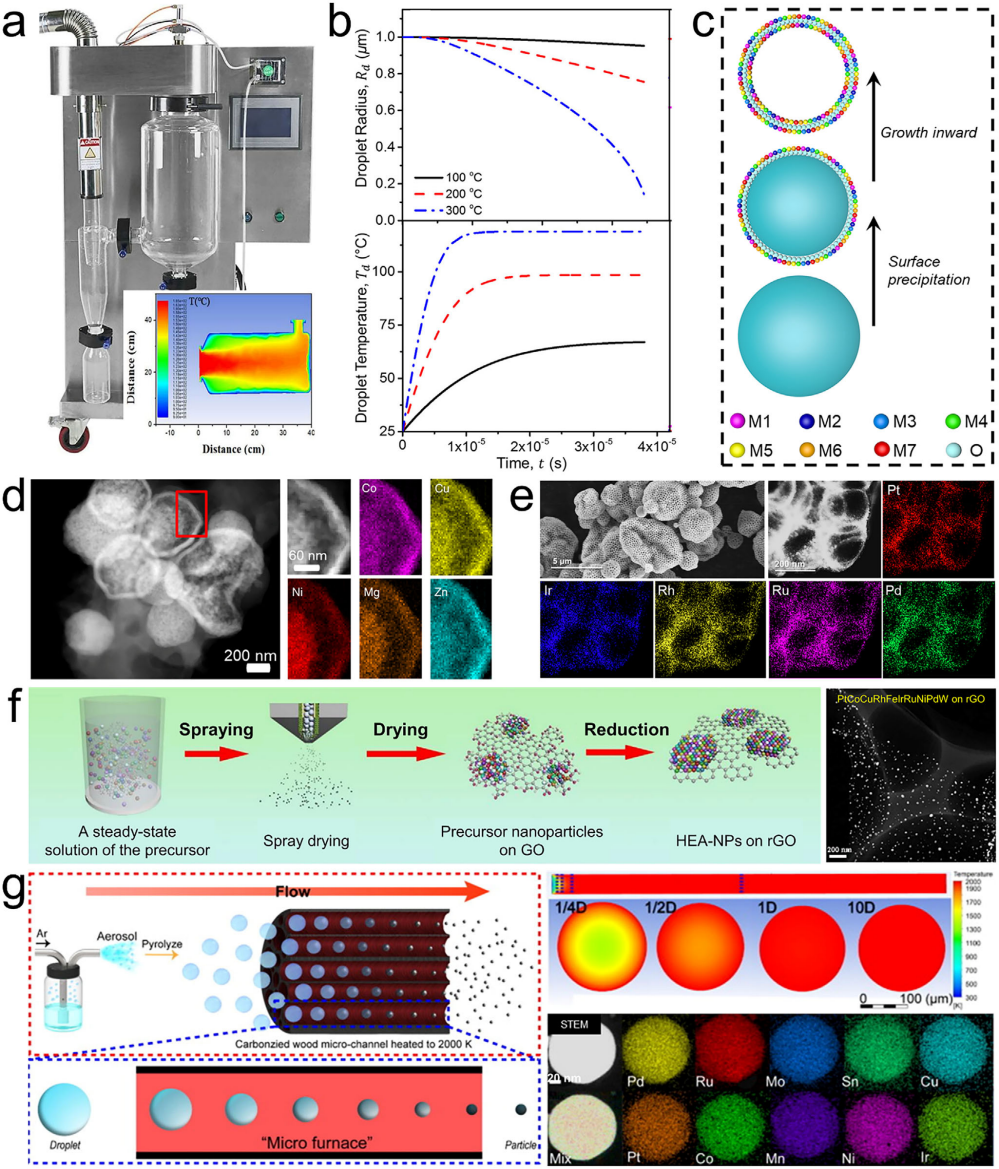
Spray drying process. (a) Spray drying reactor and its internal heating environment. Reproduced with permission [156]. Copyright 2022, American Chemical Society; (b) Microdroplet evaporation curves. Reproduced with permission [163]. Copyright 2017, American Chemical Society; (c) High‐entropy material formation mechanism via droplet‐to‐particle process; (d) (MgCoNiCuZn)O high‐entropy oxide. Reproduced with permission [167]. Copyright 2018, Springer Nature; (e) Microporous PtIrRhRuPd high‐entropy alloy. Reproduced with permission [168]. Copyright 2022, American Chemical Society; (f) Formation of supported high‐entropy alloy nanoparticles. Reproduced with permission [156]. Copyright 2022, American Chemical Society; (g) A modified spray drying process using carbonized wood micro‐channels heated to 2000 K, the temperature profile, and the resulting 10‐element high entropy alloy. Reproduced with permission [169]. Copyright 2020, Elsevier.

Spray drying is a scalable and continuous technique that enables rapid solvent evaporation and precursor solidification through atomization and convective heat transfer. In a typical process, a liquid precursor solution or suspension is atomized into micron‐sized droplets using a nozzle and then dried by contact with hot gas (Figure 10a) [156]. The liquid precursor also can be atomized by other types of aerosol generators (such as ultrasonic atomizer or air collision nebulizer) and carried into a tube furnace using a carrier gas, which enables a higher reaction temperature [157]. In the droplet‐to‐particle reaction process, each aerosol droplet serves as a microreactor. The rapid evaporation of the solvent drives the solute to quickly reach supersaturation, leading to the formation of spherical solid particles. This method is most commonly used for the synthesis of oxides, particularly for the amorphous oxides such as silica and alumina [158]. At the same time, it can also be applied to the fabrication of other ceramic materials, carbon materials, porous frameworks, and composites [159, 160]. The spray drying process provides flexible control over particle morphology and naturally favors the formation of either hollow or solid spherical structures. By incorporating soft templates, hard templates, or other functional particles into the precursor, a wide range of architectures can be achieved, such as mesoporous, microporous, core–shell, yolk–shell structures, etc [161, 162].

The rapid evaporation of droplets during the spray drying process provides favorable kinetic conditions for the non‐equilibrium synthesis of metastable and high‐entropy nanomaterials. For example at 300°C, the evaporation of a 1 µm droplet takes only about 4 × 10^−5^ s, corresponding to an effective heating rate around 1 × 10^7^ K/s (Figure 10b) [163]. Figure 10c illustrates the formation mechanism of the metastable and high‐entropy materials in this process. Under such rapid droplet‐to‐particle conversion conditions, even if the components in the precursor are thermodynamically immiscible, they have insufficient time to undergo phase separation and are kinetically stabilized into a single‐phase, supersaturated solid solution. Meanwhile, because evaporation occurs at the droplet surface, the solute at the interface reaches supersaturation first, initiating outward‐to‐inward growth and frequently resulting in hollow particle structures. This approach enables flexible doping and facilitates the synthesis of a wide range of functional inorganic materials, such as a (NiCe)O_2_ solid solution hollow nanostructure with up to 25 mol% Ni [164], Nb‐doped TiO_2_ microspheres for solar catalysis [165], and Cu‐M(M = Fe, Mg, Co, Al) bi‐metallic MOFs for CO_2_ capture [166]. Meanwhile, a hollow microsphere structured (MgCoNiCuZn)O rock‐salt high‐entropy oxide was also fabricated in this case, and showed excellent properties for reversible lithium storage (Figure 10d) [167]. In addition, spray drying can also be employed to synthesize porous high‐entropy alloy particles. In a typical approach [168], aqueous solutions of five noble metal chlorides were mixed with polymer latex particles to form a homogeneous suspension. During spray drying, atomization generated droplets containing both dissolved metal precursors and dispersed polymer beads. Rapid solvent evaporation transformed these droplets into hybrid spheroidal microparticles, in which the organic latex spheres were embedded within an inorganic matrix (Figure 10e). Subsequent thermal annealing under an inert atmosphere decomposes the polymer templates, forming mesoporous or macro‐porous high‐entropy alloy microparticles. The pore size and specific surface area could be tuned by selecting different types of polymer templates, such as polystyrene (PS) or polymethylmethacrylate (PMMA) beads, resulting in optimized catalytic performance for CO oxidation.

Combined with H_2_ thermal reduction post‐treatment, the spray drying process can also be used to synthesize supported high‐entropy alloy nanoparticles (Figure 10f) [156]. In this method, aqueous solutions of multiple metal chlorides are mixed with a suspension of carbon‐based supports (such as graphene oxide, carbon nanofibers, or multiwalled carbon nanotubes) to form a homogeneous precursor mixture. This suspension is then atomized and rapidly dried, yielding precursor nanoparticles in which metal salts are uniformly dispersed on the support. Subsequent thermal decomposition in a H_2_‐containing atmosphere at moderate temperatures (300–850°C) leads to the formation of well‐dispersed, single‐phase alloy nanoparticles. This approach showed excellent versatility, allowing the synthesis of high‐entropy alloys with up to ten elements (PtCoCuRuNiFeIrRhPdW) and compatibility with various support materials. Notably, the PtCoCuRuNiFe demonstrated outstanding electrocatalytic performance in the methanol oxidation reaction, showing 5–8 times higher mass and specific activities compared to commercial Pt/C catalysts.

A modified droplet‐to‐particle synthesis method was developed by coupling aerosol spray with a high‐temperature (∼2000 K) carbonized wood micro‐channel reactor (Figure 10g) [169]. The reactor was fabricated by carbonizing lightweight balsa wood, whose naturally aligned vessel channels (∼200 µm in diameter) serve as micron‐scale flow paths for aerosol droplets. These channels are rapidly heated by Joule heating, achieving uniform temperatures up to 2000 K with energy conversion efficiencies exceeding 90%. Upon entering the reactor, precursor droplets experience ultrafast heating and residence times of only ∼16 ms, allowing for rapid solvent evaporation, salt decomposition, and in situ formation of alloy or oxide nanoparticles. This high‐temperature, short‐time environment is critical for enabling homogeneous mixing of multiple immiscible elements, while simultaneously suppressing nanoparticle growth, agglomeration, and phase segregation. The method successfully synthesized high‐entropy alloys with complex compositions without H_2_ heating post‐treatment, such as PdRuMoSnCuPtCoMnNiIr (Figure 10g), as well as (ZnMnCoCuNi)O high‐entropy oxide, all with uniform element distribution and single‐phase solid solution structures. Unlike conventional tube furnaces (<1500 K) with temperature gradients in the radial direction and heating rates limited by conduction from the wall to the centerline of the reactor tube, the wood‐based microreactor offers superior thermal response and precise residence time control (by tuning channel length) for continuous processing, making it a more versatile non‐equilibrium synthesis method.

### 3.7 Mechanochemical Synthesis

**FIGURE 11:**
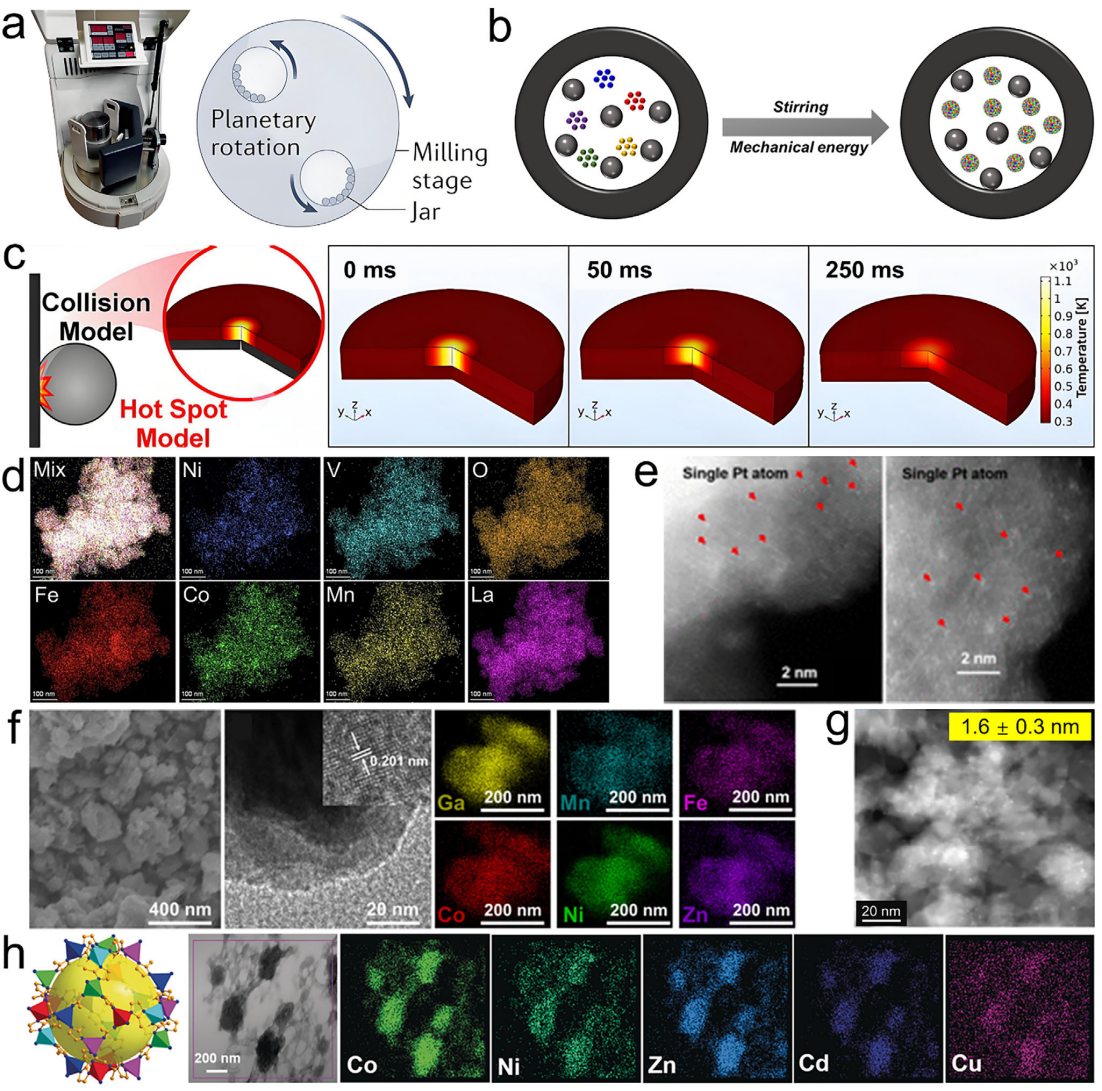
Mechanochemical synthesis. (a) Image of a planetary mill mechanochemical synthesis device. Reproduced with permission [172]. Copyright 2023, Springer Nature; (b) Schematic of high‐entropy materials formation in the mechanochemical process; (c) Hot spot and the simulated temperature profile within the hot spot at 0, 50, and 250 ms after collision. Reproduced with permission [173]. Copyright 2020, Elsevier; (d) La(NiVFeCoMn)O_3_ high‐entropy perovskite oxide. Reproduced with permission [174]. Copyright 2024, Wiley‐VCH; (e) Single‐atom Pt in (NiMgCuZnCo)O high‐entropy rock‐salt oxide. Reproduced with permission [175]. Copyright 2019, American Chemical Society; (f) GaMnFeCoNiZn high‐entropy alloy. Reproduced with permission [184]. Copyright 2025, Cell Press; (g) Supported Pt‐based high‐entropy alloy nanoparticles. Reproduced with permission [185]. Copyright 2026, Elsevier; (h) CoNiZnCdCu ZIF high‐entropy MOF. Reproduced with permission [186]. Copyright 2019, Wiley‐VCH.

Mechanochemical reactions refer to matter transformations driven by the direct absorption of mechanical energy. Typically, mechanochemical synthesis is a solid‐state reaction that is activated by mechanical energy rather than external heating [170, 171]. In high‐energy ball milling, for example, particles undergo repeated impact, shear, and compression, which continually fracture and cold‐weld the solids and generate fresh reactive interfaces (Figure 11a) [172]. Because the precursors are separated powder solids and the reactions proceed through direct solid–solid contact to achieve phase change, mechanochemistry is generally viewed as a top‐down strategy. In this respect, it differs from the bottom‐up based non‐equilibrium methods discussed earlier in this review, which achieve atomic‐level uniform mixing in a precursor and aim to maintain the homogeneous mixing in solid products by an instantaneous material formation process. However, mechanochemistry is broadly recognized as a non‐equilibrium synthesis route because mechanical impacts create extreme, highly localized thermal–mechanical environments inside the solid that cannot be accessed through conventional heating. During high‐energy ball milling, collisions between the milling ball, powder bed, and vessel wall generate microscopic “hot spots” that experience rapid pressure buildup and sharp heating/cooling. According to the collision–heat‐decay model (Figure 11c) [173], a single impact at typical milling speeds (3–5 m/s) dissipates 0.1–0.3 J into a confined volume, which can raise the temperature of the central contact zone to 1000–1120 K within 20–30 µs. Thus, this corresponds to an effective heating rate on the order of 10^7^–10^8^ K/s. After the collision ends, the temperature drops through conduction into the surrounding powder and steel wall, and simulations show that the hot spot cools below the reaction‐relevant regime within 10–40 ms, corresponding to cooling rates of 10^5^–10^6^ K/s. Such ultrafast heating/quenching is a hallmark of non‐equilibrium processing compared to long‐term heating in conventional solid‐state synthesis. The peak temperatures (>1000 K), pressures approaching the GPa range, and microsecond to millisecond lifetimes collectively provide sufficient driving force to overcome elemental immiscibility barriers and break chemical bonds, enabling a general synthesis method for metastable and high‐entropy nanomaterials with a diverse material species of ceramics, alloys, MOFs, and other coordination polymers (Figure 11b).

For example, mixing single‐metal oxides (NiO, MnO, VO_2_, Fe_2_O_3_, Co_2_O_3_, La_2_O_3_) and subjecting them to high‐energy ball milling for just 2 h can yield single‐phase high‐entropy La(NiVFeCoMn)O_3_ perovskite oxide (Figure 11d) [174], much higher activity as a CO oxidation catalyst compared to the corresponding LaMO_3_ perovskite oxide. Similarly, high‐energy ball milling also can in situ incorporate noble metal Pt into the high‐entropy (NiMgCuZnCo)O rock‐salt lattice and achieve atomic dispersion (Figure 11e), yielding a high‐performance catalyst for CO_2_ hydrogenation to CO [175]. By selecting different solid precursors, mechanochemical synthesis can produce a diverse range of ceramic materials. For example, using five metal chlorides as the metal sources together with urea as a nitrogen donor enabled the formation of (VCrNbMoZr)N high‐entropy metal nitride [176]; various high‐entropy metal sulfides can also be obtained by long‐duration ball milling (60 or 110 h) using metal sulfides and sulfur powder as the starting precursors [177]; Similarly, high‐entropy carbides [178], high‐entropy hydroxides [179], rutile‐type high‐entropy fluorides [180], perovskite‐structured high‐entropy halides (Br, I) [181], and layer‐structured high‐entropy phosphate Dittmarite analogues [182] can all be synthesized via mechanochemical routes. These materials have demonstrated excellent performance across a variety of applications, including Li‐ion batteries, thermal insulation, OER electrocatalysis, and X‐ray flat‐panel detectors. Furthermore, the mechanochemistry synthesis can also create mesopore structures in high‐entropy ceramics by a nonhydrolytic sol−gel strategy [183].

Mechanochemical methods have also been used to synthesize high‐entropy alloys using metal powders as precursors, using liquid metal Ga as a reaction medium during mechanically driven alloying (Figure 11f) [184]. Because Ga is liquid near room temperature and can partially dissolve many transition metals, it creates a fluid interface where metal atoms continuously dissolve, mix, and reprecipitate under mechanical agitation. Negative mixing enthalpies between Ga and various metals further lower the barrier for alloying, while the liquid environment provides a much faster diffusion pathway than solid‐state milling alone. As milling proceeds, dissolved metals homogenize within Ga and gradually nucleate into multicomponent alloy clusters, eventually yielding a single‐phase high‐entropy alloy. This Ga‐mediated approach enables room‐temperature synthesis of 5–9 element high‐entropy alloys using only simple mechanical equipment and very low energy input, significantly reducing reaction time and cost. In addition, mechanochemical synthesis can also be combined with support engineering to produce ultra‐small high‐entropy alloy nanoparticles with exceptional stability [185]. First, ball milling was used to generate sub‐nanometer multi‐metal acetylacetonate clusters, which were subsequently immobilized onto in situ–formed Mg(OH)_2_ seeds. After calcination, this mechanochemistry‐assisted pathway yielded Pt‐based high‐entropy alloy nanoclusters uniformly confined on MgO nano‐islands (Figure 11g). The nanoscale confinement effectively suppresses particle migration and Ostwald ripening, maintaining cluster sizes below 2–3 nm even after reduction at 800°C, where unsupported counterparts rapidly sinter to >10 nm. The confined high‐entropy alloy nanoparticle exhibits markedly improved electronic uniformity and nearly complete noble‐metal dispersion, enabling high propane conversion and long‐term durability during dehydrogenation.

More importantly, the mechanochemical synthesis provides a route to create high‐entropy MOFs. For example, vigorous ball milling of ZnO, CuO, CdO, Ni(OAc)_2_, and Co(OAc)_2_ with an excess of 2‐methylimidazole organic linker for 120 min yielded a phase‐pure high‐entropy ZIF after simple washing (Figure 11h) [186]. This material showed significantly higher yields than the corresponding single‐metal ZIFs in the catalytic conversion of CO_2_ into carbonates. Similarly, by using 2,5‐dihydroxyterephthalic acid (DHTA) as an organic ligand, a high‐entropy ZnMgNiCoCu MOF‐74 was fabricated [187]. Furthermore, high‐entropy Prussian blue analogues (PBAs), an inorganic coordination polymer analogous to MOFs such as the K(MgMnFeCoNi)Fe(CN)_6_, can also be synthesized through simple ball‐milling and water‐washing. This high entropy PBA exhibited higher capacitance than the corresponding single‐metal Prussian blue analogues [188]. These examples demonstrate that mechanochemical synthesis is a versatile and powerful route for producing wide high‐entropy materials for diverse applications.

### 3.8 Other Non‐Equilibrium Synthesis Methods

#### 3.8.1 Ultrasonication Synthesis

**FIGURE 12:**
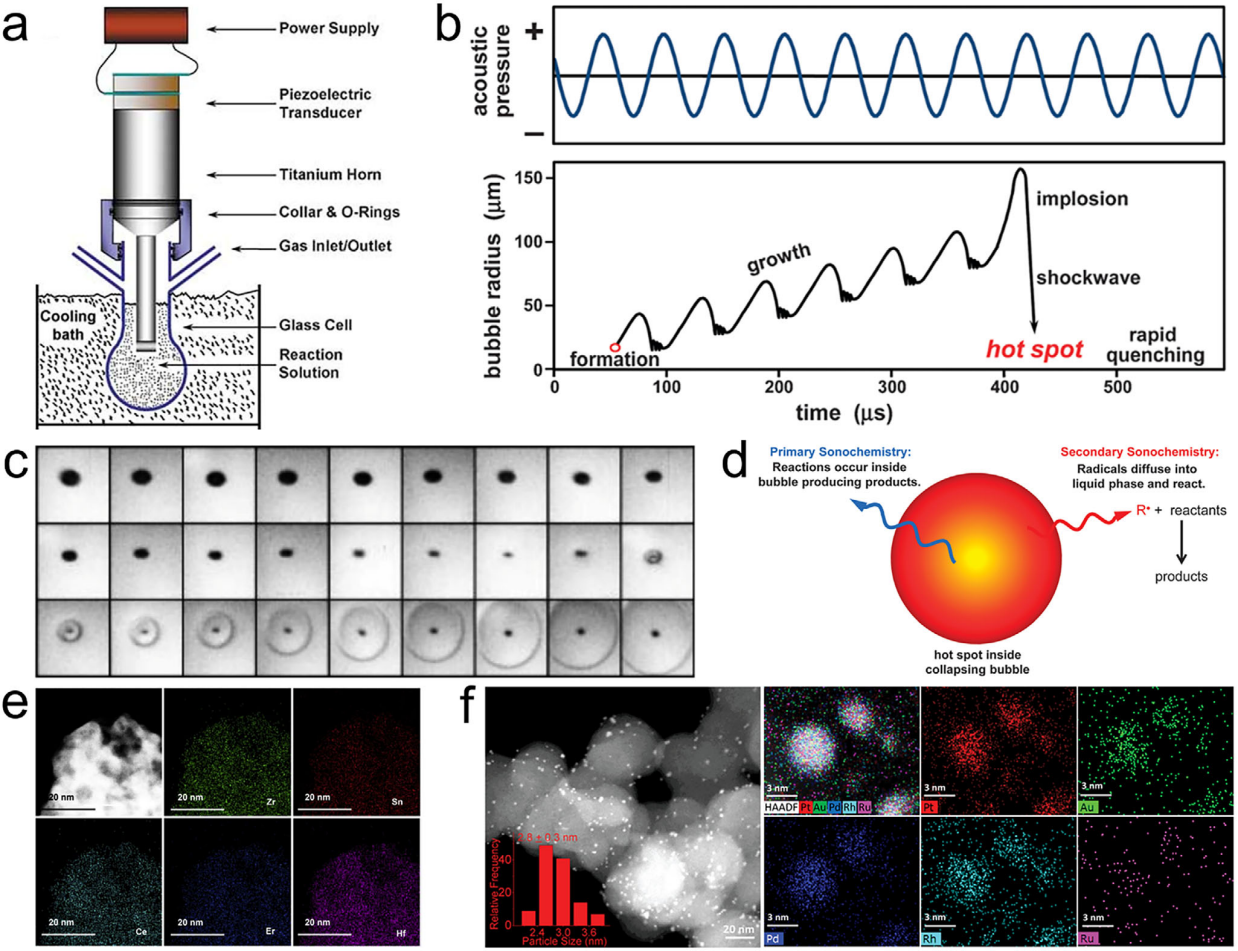
Ultrasonic synthesis. (a) Ultrasonic synthesis apparatus with a high‐intensity ultrasonic horn. Reproduced with permission [189]. Copyright 2010, Wiley‐VCH; (b) Schematic of the acoustic cavitation process, (c) a spherical shock wave launched from a collapsing bubble. The shock wave can reach 60 kbar with a velocity of 4 km/s, and (d) primary sonochemistry and secondary sonochemistry driving nanomaterial formation. Reproduced with permission [190]. Copyright 2013, Royal Society of Chemistry; (e) (ZrSnCeErHf)O_2_ high‐entropy fluorite oxide. Reproduced with permission [193]. Copyright 2022, Cell Press; (f) PtAuPdRhRu/carbon high‐entropy alloy nanoparticles. Reproduced with permission [194]. Copyright 2019, Wiley‐VCH.

Ultrasonic synthesis offers a unique route for fabricating nanostructured materials under ambient pressure and near‐room‐temperature conditions. Unlike conventional methods that often require high temperatures, high pressures, or long reaction durations, this technique uses high‐intensity ultrasound, typically in the 20 kHz to MHz range, delivered through a piezoelectric transducer and titanium horn to inject acoustic energy into a liquid medium (Figure 12a) [189]. The resulting reaction environment is highly localized and transient, enabling rapid energy delivery without bulk heating. Thus, ultrasonic synthesis is not only cost‐effective and experimentally straightforward, but also easily scalable, making it an attractive platform for laboratory research and potential industrial implementation.

The core mechanism of ultrasonic chemistry is acoustic cavitation, the formation, growth, and sudden collapse of microbubbles in a liquid under alternating acoustic pressure fields (Figure 12b) [190]. As high‐intensity ultrasound propagates, it induces cycles of compression and rarefaction that generate dynamic tensile stresses. During rarefaction, small gas nuclei or impurities expand over successive cycles through rectified diffusion. Once these bubbles reach a critical size, they become unstable and undergo inertial collapse, concentrating energy into a microscopic volume. This collapse produces a transient “hot spot” characterized by extremely high local temperatures and pressures, accompanied by shockwave generation and rapid quenching of bubble contents in the surrounding liquid. Real‐time imaging of this process (Figure 12c) reveals the sequential evolution of cavitation, including bubble formation, expansion, and collapse [191]. At the final stage, the implosion emits shockwaves reaching pressures up to 60 kbar and velocities of 4 km/s. This intense mechanical energy initiates chemical reactions and physical transformations in the surrounding medium, giving rise to two sonochemical pathways (Figure 12d). Primary sonochemistry occurs inside the bubble, where volatile organometallics undergo bond cleavage, generating reactive metal atoms or radicals that nucleate into nanoparticles upon entering the liquid. Secondary sonochemistry takes place in the surrounding solution, where radicals like •OH and •H diffuse outward to trigger redox or polymerization reactions, even with non‐volatile precursors like metal salts. Together, these coupled processes create a highly reactive and tunable chemical environment, enabling the synthesis of diverse nanomaterials including metals, ceramics, and carbon materials.

Importantly, acoustic cavitation provides an extreme and transient microenvironment with localized temperatures exceeding 5000 K, pressures over 1000 atm, and heating/cooling rates greater than 10^10^ K/s [192], offering ideal conditions for the non‐equilibrium synthesis of metastable phases and high‐entropy materials. For example, a 15‐min ultrasonic treatment of a mixed aqueous solution of metal chlorides and NaOH at 20 kHz under ambient conditions directly produced fluorite‐structured (ZrSnCeErHf)O_2_ high‐entropy nanocrystals (Figure 12e), without the need for high‐temperature sintering. The resulting materials possessed high surface area and abundant oxygen vacancies and exhibited excellent CO oxidation catalytic performance when loaded with Pd nanoparticles [193]. Also, ultrasonication‐assisted synthesis at ambient conditions enabled the formation of uniform PtAuPdRhRu high‐entropy alloy nanoparticles (∼2.8 nm) supported on carbon (Figure 12f), which showed better electrocatalytic performance for the HER reaction than a commercial Pt/C catalyst [194]. Similarly, BaSr(ZrHfTi)O_3_ and BaSrBi(ZrHfTiFe)O_3_ high‐entropy perovskite oxides [195], as well as AuPdPtRhRu high‐entropy alloy nanoparticles [196] were fabricated by this method.

#### 3.8.2 Ultrafast Photoflash

**FIGURE 13:**
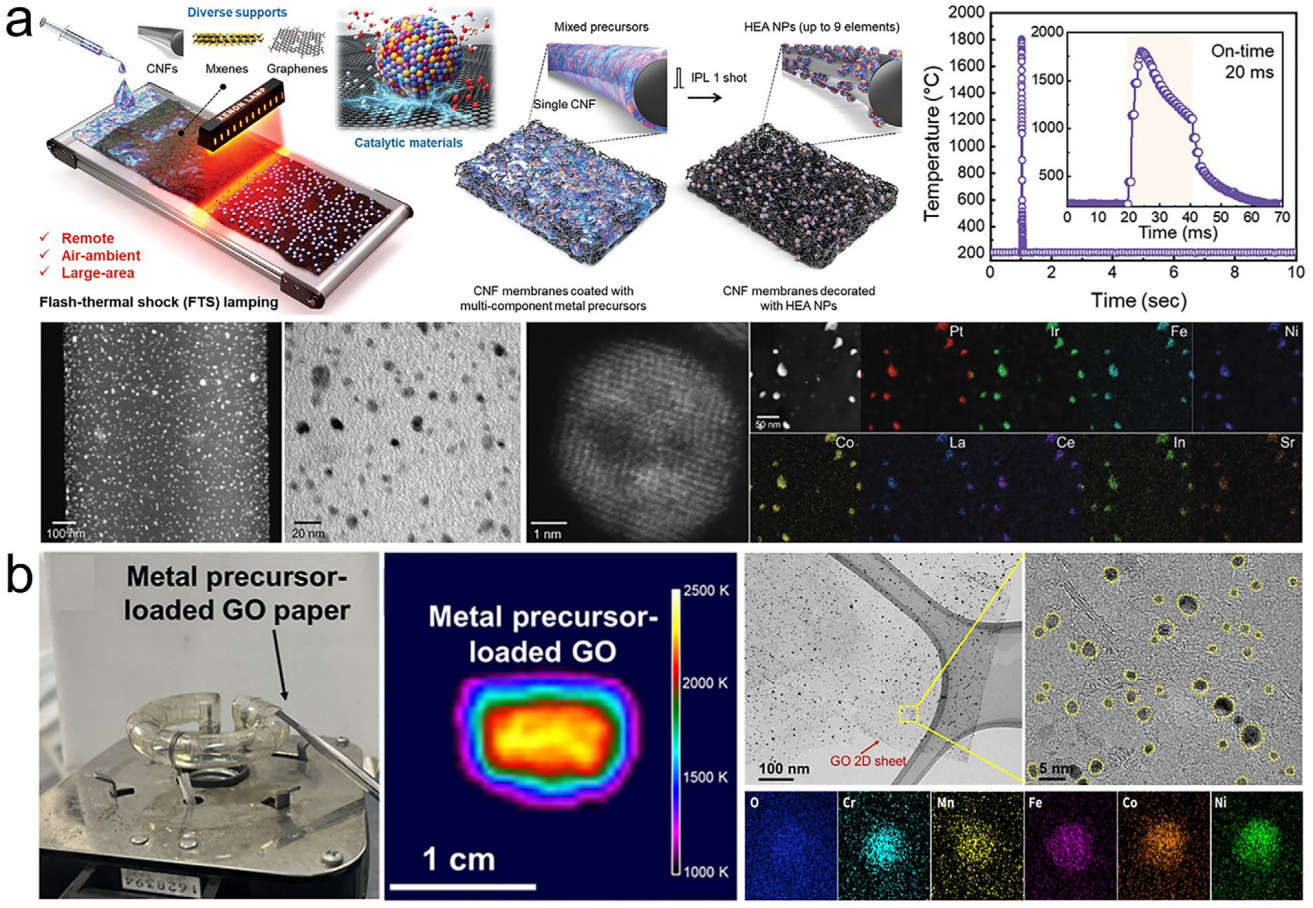
Ultrafast photoflash. (a) Illustration of a potential roll‐to‐roll ultrafast xenon lamp photoflash process to fabricate supported high‐entropy alloy nanoparticles, heating/cooling rates, and a typical PtIrFeNiCoLaCeInSr/carbon nanofiber produced. Reproduced with permission [198]. Copyright 2023, Wiley‐VCH; (b) Ultrafast xenon lamp photoflash synthesis of supported high‐entropy oxide nanoparticles, the temperature profile, and the typical (CrMnFeCoNi)_3_O_4_ spinel oxide product. Reproduced with permission [199]. Copyright 2025, American Chemical Society.

Photo‐thermal effects arise from energy exchange between incident photons and electrons in materials. The photo‐excited electrons then rapidly transfer their energy to the atomic lattice, resulting in localized and transient heating of the illuminated regions [197]. This mechanism, based on photon–electron coupling and phonon‐mediated energy conversion, is particularly effective in carbon‐based substrates such as carbon nanofibers (CNFs), MXenes, or graphene, which exhibit high light absorption and thermal responsiveness. Building on this principle, ultrafast photoflash synthesis has emerged as a powerful approach for the rapid, scalable, and ambient‐condition fabrication of nanomaterials. In this method, millisecond‐scale, high‐intensity light pulses, typically from a xenon (Xe) flash lamp, irradiate substrates pre‐coated with multicomponent metal precursors. The absorbed photon energy induces a flash‐thermal shock, causing the substrate surface to reach temperatures exceeding 1800°C within ∼20 ms, followed by cooling rates greater than 10^4^ K/s (Figure 13a) [198]. Such highly non‐equilibrium thermal conditions are especially favorable for the formation of metastable phases and high‐entropy materials. The rapid temperature spike initiates precursor decomposition and the localized fusion of transient metal droplets, which are quickly quenched, trapping the immiscible elements in well‐mixed solid‐solution nanoparticles. Thus, highly dispersed alloy nanoparticles can be formed on carbon materials, such as a PtIrFeNiCoLaCeInSr/carbon nanofiber structure that incorporates many immiscible elements with non‐FCC crystal structures (Figure 13a) [198]. PtIrFeNiCoCe prepared by this approach demonstrated high activity and excellent stability over 5000 cycles in water splitting, including both HER and OER reactions. Importantly, this process is amenable to scalable manufacturing. The one‐step photoflash irradiation eliminates the need for prolonged thermal treatments, vacuum systems, or conductive supports. Moreover, the wide‐area beam of the Xe flash lamp makes this method compatible with roll‐to‐roll processing, offering a practical route toward continuous production of nanomaterials supported on carbon‐based substrates. However, the scalability here primarily refers to producing large areas. The mass loading achievable is typically on the order of ∼1 g m^−2^, meaning that while thin membranes can be uniformly decorated with nanoparticles over large areas at high rates, producing large masses or volumes of HEA nanomaterials by this approach remains challenging.

Similarly, a simple photoflash synthesis method was developed to prepare high‐entropy oxide nanoparticles using only a commercial xenon (Xe) photoflash lamp as the energy source (Figure 13b) [199]. This approach exploits the photothermal properties of graphene oxide (GO), which undergoes exothermic disproportionation upon illumination. The resulting heat rapidly drives the decomposition and oxidation of multi‐metal precursors, forming well‐dispersed HEO nanoparticles within tens of milliseconds. The process features ultrafast heating (∼10^6^ K/s) and cooling (∼10^5^ K/s) rates, with the local temperature peaking above 2250 K and sustained over 1000 K for ∼30 ms. The particle size could be tuned from a few nanometers to tens of nanometers by adjusting precursor concentrations and flash parameters. Compared to flash Joule heating, which requires expensive pulsed power supplies, this photoflash method is cost‐effective and contactless for loading nanoparticles on the substrates that are strong absorbers of the light, mostly carbon‐based substrates. It can also form much smaller nanoparticles than flash Joule heating (CrMnFeCoNi)_3_O_4_ high‐entropy spinel oxide generated by this approach exhibited excellent OER performance and long‐term stability comparable to conventionally annealed counterparts, highlighting the potential of photoflash synthesis for fast, low‐cost production of functional nanomaterials.

#### 3.8.3 Electrical Explosion

**FIGURE 14:**
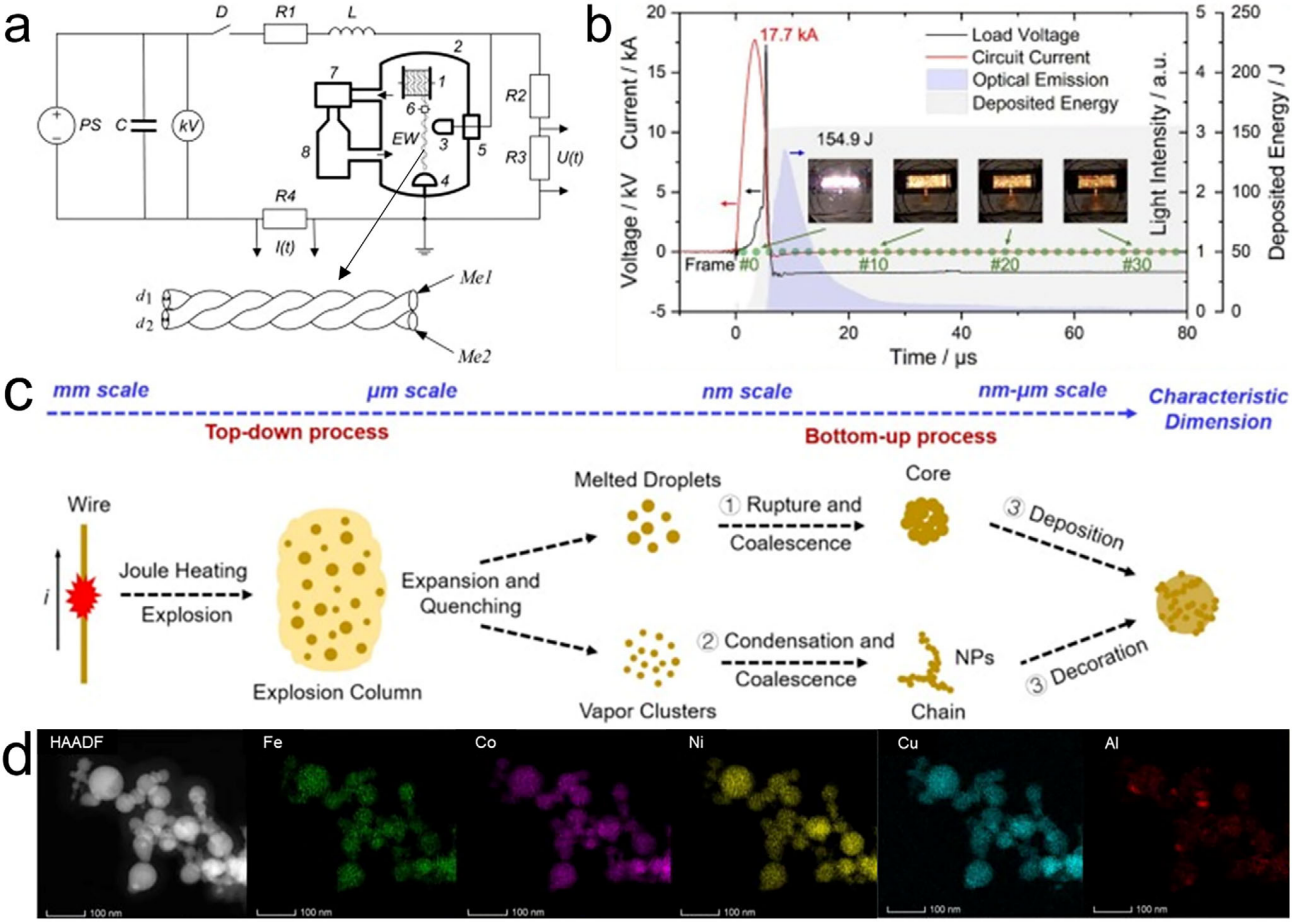
Electrical explosion of wires. (a) Circuit diagram of the apparatus employed for the explosion of twisted wires. Reproduced with permission [201]. Copyright 2016, Elsevier; (b) Voltage, current, and light‐intensity waveforms and typical backlit images (under U = 9.0 kV condition). The images, with exposure time below 1µs, are synchronized with the discharge process, and (c) nanoparticle formation mechanisms. Reproduced with permission [203]. Copyright 2025, Elsevier; (d) FeCoNiCuAl high‐entropy alloy nanoparticles. Reproduced with permission [205]. Copyright 2024, American Institute of Physics.

Electrical explosion of wires is a physical vapor‐based technique in which a metal wire is rapidly heated to explosion by a high‐density current pulse (10^6^–10^9^ A/cm^2^). The intense Joule heating causes the wire to melt, vaporize, and partially ionize into a plasma state within microseconds, which can produce metastable or composite nanoparticles with tunable size and phase composition [200]. The electrical explosion of wires (EEW) is carried out in a high‐voltage RLC discharge circuit coupled with an inert gas chamber (Figure 14a) [201]: In this process, metal wires are rapidly heated and vaporized by a high‐current pulse (typically 20–30 kV), leading to an explosive expansion and quenching into nanoparticles. The produced particles are transported by an inert gas stream and collected downstream. This method provides high productivity (up to 200 g/h), yields nanoparticles with sizes of 20–100 nm, and operates with relatively low energy consumption (∼25 kWh/kg) [202].

Figure 14b illustrates the dynamic process of electrical explosion [203]. At the onset of discharge, both current and voltage rise simultaneously with negligible resistance change, corresponding to the wire heating phase. The current peaks at 17.7 kA at 3.40 µs and then drops rapidly. Meanwhile, the voltage reaches 17.3 kV at 5.35 µs, indicating electrical breakdown. This is followed by a sharp drop in both voltage and resistance, due to the formation of a low‐resistance plasma channel. The optical emission intensity peaks around 6 µs. Time‐resolved images reveal that the wire undergoes sequential phase transitions, melting, vaporization, and ionization, characterized by distinct emission colors. The explosion products are expelled as a high‐velocity jet, initially propagating at 0.81 km/s into the surrounding medium. A noticeable decay occurs over a 100 µs jetting period, during which the plasma jet mixes with the ambient gas. In this process (Figure 14c), the resulting high‐temperature wire‐explosion column contains both unvaporized liquid droplets and vaporized metal atoms and molecules. The explosion products rapidly expand and undergo quenching. The quenched products originate from two distinct physical pathways. First, the unvaporized droplets fragment into submicron molten droplets, which subsequently solidify via a droplet‐to‐particle mechanism, forming relatively large particles. Second, vaporized metal clusters nucleate and grow through a gas‐to‐particle pathway, yielding much smaller nanoparticles. In some cases, the smaller nanoparticles deposit onto the surface of larger molten‐derived spheres, resulting in supported or core–shell‐like structures.

Thus, the intense electrical explosion process provides extremely high heating and rapid quenching rates (10^10^–10^12^ K/s) [204], creating highly non‐equilibrium conditions for preparing metastable and high‐entropy nanomaterials. For example, it enabled the integration of immiscible couples of Cu─Ag, Сu─Pb and Al─Pb in binary metallic nanoparticles [201]; FeCoNiCuAl high‐entropy alloy nanoparticles were also produced and demonstrated excellent electromagnetic wave absorption properties (Figure 14d) [205]; (FeCoNiCrCu)O high‐entropy rock‐salt and (FeCoNiCrTi)_3_O_4_ spinel oxides were also produced by electrical explosion of wires in an O_2_‐containing atmosphere [206].

#### 3.8.4 Leidenfrost Droplet Explosion

A Leidenfrost droplet refers to a liquid droplet placed on a surface heated well above its boiling point, typically exceeding the so‐called Leidenfrost temperature. Under such conditions, the bottom layer of the droplet undergoes rapid vaporization, forming a continuous cushion of vapor that lifts and insulates the droplet from direct contact with the hot surface. This vapor layer drastically reduces heat transfer by conduction and alters both the evaporation rate and hydrodynamic behavior of the droplet. As a result, Leidenfrost droplets exhibit unique dynamic phenomena, including self‐propulsion, shape oscillations, and prolonged evaporation lifetimes [207]. These behaviors have attracted growing interest not only in fundamental fluid physics but also in practical nanomaterial fabrication. For example, evaporating Leidenfrost droplets floating on a hot surface (∼270°C) can act as microscale reactors to synthesize ∼4 nm gold nanoparticles [208], while the underwater Leidenfrost effect has been used to produce ∼50 nm metastable hollow zinc peroxide (ZnO_2_) nanospheres [209]. Furthermore, when the Leidenfrost droplet is relatively large and contains trace levels of contaminants or suspended particles, an explosive disintegration can occur at the final stage of evaporation [210]. As the droplet gradually shrinks, these impurities accumulate at the liquid–air interface and eventually form a thin solid shell. When the vapor layer beneath the droplet thins to a critical level comparable to the particle size (∼1 µm), the shell loses support and locally collapses, leading to transient contact between the hot substrate and the liquid core. This sudden contact triggers rapid nucleation and boiling at the interface, resulting in the explosion of the droplet.

**FIGURE 15:**
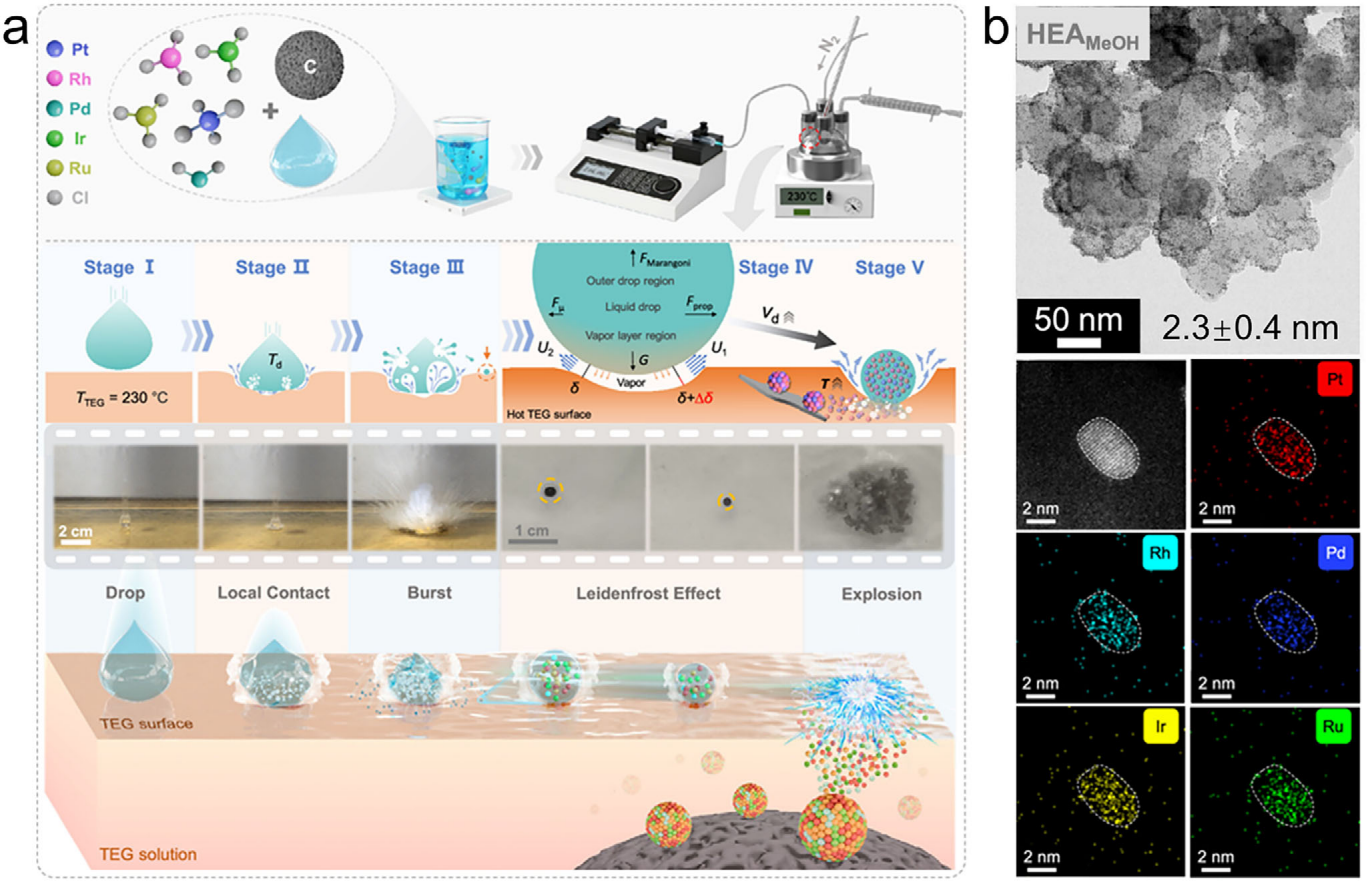
Leidenfrost droplet explosion. (a) The synthesis device, Lidenfrost droplet explosion, and high‐entropy alloy nanoparticle formation processes; (b) PtRhPdIrRu/carbon high‐entropy alloy nanoparticles prepared with methanol as solvent. Reproduced with permission [211]. Copyright 2024, American Chemical Society.

Such Leidenfrost droplet explosion phenomenon can also create non‐equilibrium reaction environments for the synthesis of high‐entropy alloy nanomaterials [211]. As shown in Figure 15a, a suspension containing Pt, Rh, Pd, Ir, and Ru chlorides mixed with carbon black in methanol was introduced dropwise onto a triethylene glycol (TEG) pool preheated to 230°C. Upon contact (Stage I), the methanol rapidly vaporized (Stage II), leading to violent bubble formation within the droplet. This internal gas generation disintegrated the parent droplet into smaller fragments (Stage III). These secondary droplets levitated over the TEG surface, supported by a balance between the Marangoni force and gravity, and were propelled laterally due to asymmetric vapor flow beneath (Stage IV). During lateral motion, the droplets shrank as methanol evaporate, while carbon particles accumulated at the interface, eventually impeding vapor formation. When the Marangoni force could no longer sustain levitation, the droplet recontacted the hot surface, triggering rapid methanol vaporization and a sharp internal pressure rise that triggered droplet explosion (Stage V). The fragments were dispersed into the hot TEG bath, where ultrafast heat and mass transfer, combined with the reducing environment of TEG, converted the precursors into high‐entropy alloy nanoparticles. This process produced ∼2.3 nm PtRhPdIrRu/carbon high‐entropy alloy nanoparticles (Figure 15b), which exhibited excellent electrocatalytic performance for HOR and HER reactions.

## 4 Summary and Outlook

This review systematically highlighted a suite of non‐equilibrium synthesis strategies that overcome the intrinsic thermodynamic constraints limiting conventional solid‐state material design. Unlike classical equilibrium‐based approaches—where nucleation and growth are governed by slow atomic diffusion over extended timescales—these non‐equilibrium methods operate within drastically shortened temporal windows, ranging from milliseconds down to nanoseconds. These rapid thermal transients fundamentally alter phase formation pathways by kinetically trapping atomic configurations before long‐range diffusion or phase separation can take place. As summarized in Table 1, these non‐equilibrium synthesis methods encompass a wide spectrum of thermal environments, kinetic profiles, and reaction media. Achievable temperatures range from modest levels around 500 K, as seen in Leidenfrost droplet explosion, to extreme conditions surpassing 10,000 K in discharge plasma and electrical explosion systems. Correspondingly, heating and cooling rates span over ten orders of magnitude from 10^3^  to 10^14^ K/s, providing access to a diverse range of time‐temperature trajectories. These techniques also operate across diverse physical phases: vapor‐phase processes dominate in flame spray pyrolysis and plasma methods; liquid‐phase strategies underpin ultrasonic and microwave synthesis; while solid‐state restructuring is typical in photoflash and Joule heating approaches. Despite their differences in energy sources and reactor design, all these methods share a unifying principle: the application of extreme thermal gradients over ultrashort times to bypass equilibrium limitations. This allows direct conversion of disordered elemental mixtures, often in vapor or liquid precursor forms, into solid‐state nanostructures, stabilizing metastable compositions and trapping otherwise immiscible elements into single‐phase high‐entropy ceramics, alloys, and MOFs.

**TABLE 1:** Summary and evaluation of non‐equilibrium synthesis methods.

Non‐equilibrium methods	Temperature (K)	Heating rate (K/s)	Cooling rate (K/s)	Phase	High‐throughput difficulty	Scalable potential
Flash Joule heating	Up to 3000	10^5^–10^6^	10^3^–10^5^	Solid	Moderate	Medium
Flame spray pyrolysis	∼2000	10^6^–10^7^	10^4^	Vapor	Easy	High
Discharge plasma	Up to 10 000	10^6^–10^9^	10^5^–10^6^	Vapor/Solid	Moderate	High
Laser ablation	Up to 4000	Up to 10^14^	Up to 10^12^	Vapor/Liquid/Solid	Easy	Medium
Microwave heating	Up to 2000	∼10^4^	∼10^4^	Liquid/Solid	Difficult	Medium
Spray drying process	500–2000	10^4^–10^7^	10^2^–10^4^	Vapor	Easy	High
Mechanochemical synthesis	∼1000	10^7^–10^8^	10^5^–10^6^	solid	Moderate	High
Ultrasonic synthesis	Up to 5000	∼10^10^	∼10^10^	Liquid	Difficult	Medium
Ultrafast photoflash	Up to 2250	∼10^6^	10^4^–10^5^	Solid	Easy	Low
Electrical explosion	Up to 10 000	10^10^–10^12^	10^10^–10^12^	Vapor	Easy	High
Leidenfrost droplets explosion	∼500	10^4^–10^5^	10^3^–10^5^	Liquid	Difficult	Low

While non‐equilibrium synthesis methods offer remarkable opportunities, they also come with inherent drawbacks that merit careful consideration. A central challenge lies in the violent and transient nature of many processes, which makes precise control of product morphology and particle size distribution, and engineering of specific crystal structures such as ordered intermetallics or complex compounds quite challenging. For example, flash Joule heating, discharge plasma, and electrical explosion of wires generate extreme temperatures and quenching rates, which promote rapid nucleation but often lead to heterogeneous particle sizes and limited control over morphology and surface chemistry. This is also the case for flame spray pyrolysis and spray drying, though the timescales are somewhat shorter. Laser ablation and ultrafast photoflash methods provide unparalleled spatiotemporal precision, but their material yields are typically low, and reproducibility may suffer when targeting bulk‐scale powder synthesis. In addition, most of these methods inherently involve intense, high‐temperature reaction environments, which make them unsuitable for synthesizing thermally sensitive materials such as organic materials and other low‐melting or volatile compounds. Ultrasonic and microwave‐based approaches operate under milder conditions, yet they are prone to uneven heating or limited penetration depths, which complicate scale‐up and may compromise product uniformity.

Despite these, the unique strengths of non‐equilibrium synthesis methods are undeniable. For example, these non‐equilibrium synthesis methods inherently offer distinct advantages for high‐throughput synthesis and industrial‐scale manufacturing. Their intrinsic ability to bypass thermodynamic limitations allows the exploration of nearly infinite compositional spaces that can provide invaluable datasets for AI‐driven optimization. Guided by rapidly advancing machine learning based approaches, these strengths can be harnessed to accelerate materials discovery, optimization, and large‐scale production. These features make non‐equilibrium synthesis methods particularly well‐suited to meet the demands of modern materials science, where speed, scalability, and data‐rich innovation are critical drivers of progress. Based on this, we propose the following outlook for the development of non‐equilibrium synthesis in the field of inorganic nanomaterials:

### 4.1 High‐Throughput Synthesis

**FIGURE 16:**
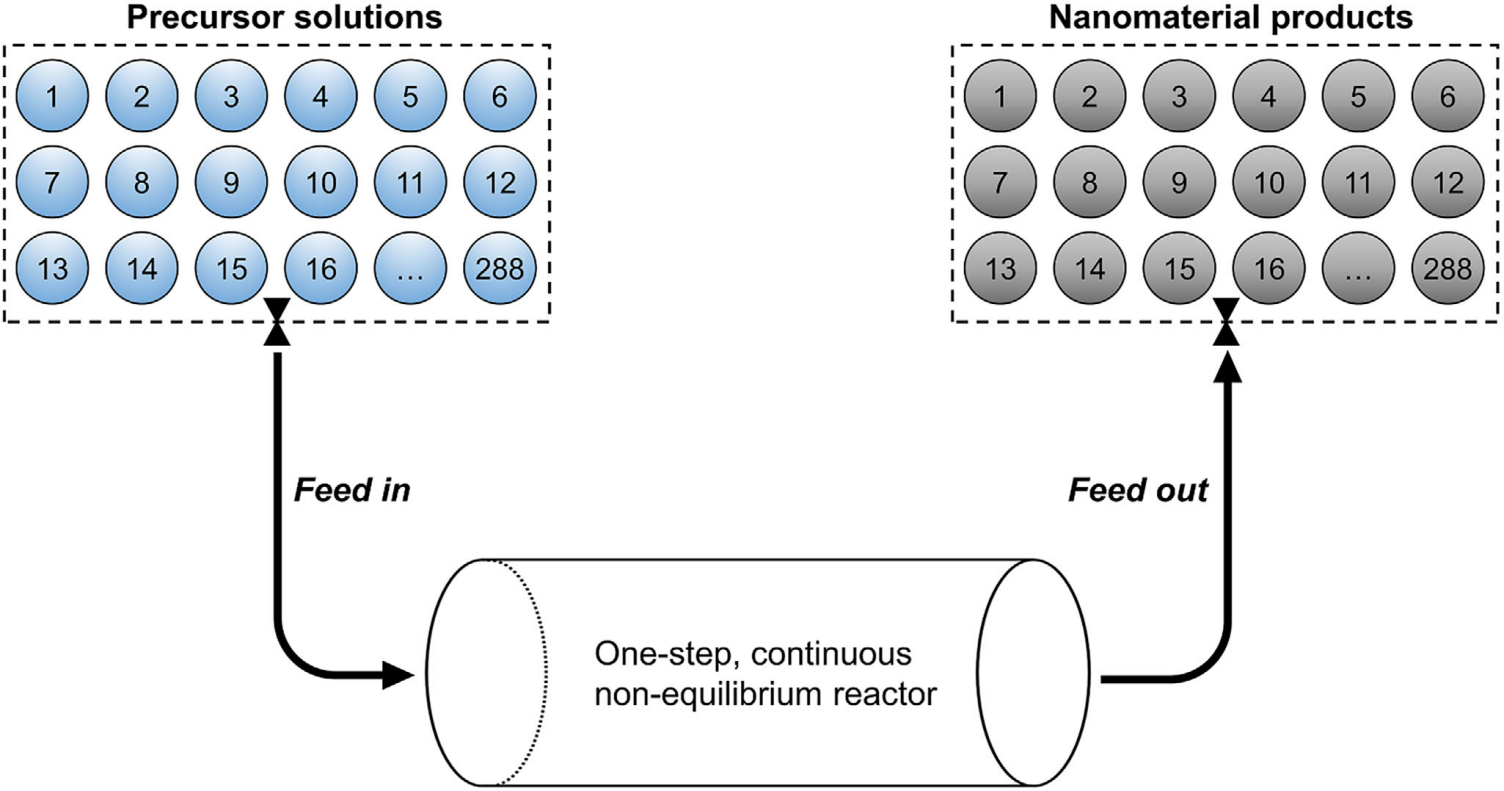
One‐step and continuous non‐equilibrium reactor for high‐throughput synthesis.

High‐throughput synthesis refers to the rapid, parallel fabrication of a large number of samples within a short timeframe. For example, in a recent study a robotic experimental platform operated autonomously for over eight days, fabricating 688 catalysts without human intervention [212]. Conventional equilibrium‐based synthesis methods, including wet‐chemical approaches like hydrothermal and sol–gel reactions, as well as solid‐state techniques such as ball milling followed by calcination, typically involve a series of time‐ and labor‐intensive steps such as stirring, heating, centrifugation, and drying. This not only increases the costs of building high‐throughput synthesis labs based on such methods [213], but also reduces the synthesis efficiency. In contrast, most of the non‐equilibrium synthesis methods discussed here enable fast and one‐step operation, well‐suited for generating numerous diverse samples in a short time. For example, by combining programmable printing with salt precursor deposition onto carbon nanofiber films followed by rapid flash Joule heating, alloy nanoparticles of many different compositions can be synthesized within minutes. This approach enables high‐throughput synthesis of alloy nanoparticles, ranging from ternary to octonary compositions [214]. Laser ablation also possesses a comparable high‐throughput synthesis capability. By ablating mixed solid powder precursors mounted on a rotating target stage, up to 40 distinct catalysts can be fabricated in one day [215]. Moreover, many vapor‐phase non‐equilibrium synthesis methods [216], including flame spray pyrolysis, spray drying process, thermal plasma, and gas‐phase laser pyrolysis, are inherently one‐step and continuous processes, providing a very simple and low‐cost route to achieve high‐throughput synthesis. In these aerosol‐based synthesis processes, different precursor solutions can be fed into the system, and then the distinct products can be directly collected downstream, with simple valve switching enabling high‐throughput production within a single reactor (Figure 16). By maintaining continuous operation and switching precursor solutions every 5 min, up to 288 different samples could be synthesized in one day. The inherently simple and one‐step nature of non‐equilibrium synthesis methods makes them particularly well‐suited for high‐throughput production. Therefore, building high‐throughput synthesis platforms based on these methods could significantly enhance the efficiency of material design and discovery.

### 4.2 Industrial Production

Bridging the gap between laboratory‐scale synthesis and industrial‐scale production, i.e., achieving true scalability, remains a fundamental challenge in nanomaterial development. Many conventional equilibrium‐based synthesis methods face serious obstacles to scale‐up, including high manufacturing costs and significant changes in physicochemical properties during upscaling. In contrast, non‐equilibrium synthesis methods, particularly those that are one‐step and operationally simple, offer intrinsic advantages for industrial translation. Several techniques, such as flash Joule heating [79], laser ablation [217], and ultrafast photoflash synthesis [198], are compatible with roll‐to‐roll manufacturing platforms, enabling continuous production at rates of several meters per minute. These provide straightforward routes to producing nanomaterials over large areas of substrates or membranes. However, it is important to note that roll‐to‐roll manufacturing and genuinely high‐yield production do not necessarily coincide. Roll‐to‐roll platforms are ideally suited for coating or decorating large‐area substrates, such as carbon papers, membranes, or flexible films, at rates of square meters or more per minute. This capability is extremely attractive for device integration, where uniform coverage over macroscopic areas is essential. Nevertheless, the actual material mass deposited per unit area is relatively low, often on the order of ∼1 g/m, so even square‐meter–scale processing translates to only gram‐level material output. As a result, while roll‐to‐roll processes enable scalability in terms of area and throughput, they are less effective when kilogram‐scale powder production or higher yield nanomaterial synthesis is required.

In contrast, vapor‐phase, one‐step continuous aerosol‐based methods, most notably flame spray pyrolysis and spray drying, are inherently designed for continuous, high‐yield powder production. These methods have been established as industrial workhorses since World War II, which can maintain product uniformity while delivering true high‐mass yields, making them more aligned with the conventional notion of scalable high‐yield manufacturing. These techniques are widely used for producing nano‐powders at massive scales, including carbon black, fumed silica, and TiO_2_. Annual production of such materials reaches millions of tons globally, with individual industrial reactors capable of generating several tons per hour [80, 81, 82, 216]. Compared to many wet‐chemical synthesis methods, which can be highly sensitive to precursor concentration and prone to producing inconsistent particle size and morphology during scale‐up, aerosol‐based gas‐phase processes exhibit robust process stability. Thanks to the continuous and modular nature of these vapor‐phase synthesis processes, they can often maintain material quality and uniformity even under increased production loads. As a result, aerosol‐based synthesis methods are no longer limited to producing low‐cost carbon black or ceramic oxides. Their inherent scalability and non‐equilibrium characteristics offer a promising pathway for the future industrial‐scale production of high‐entropy nanomaterials, particularly for large volumes of unsupported (substrate‐free) or powder‐supported nanomaterials.

### 4.3 Challenges and Opportunities in the Age of AI

The synthetic flexibility of non‐equilibrium methods in overcoming elemental thermodynamic immiscibility can dramatically expand the compositional landscape of inorganic materials. For example, in a 10‐element high‐entropy alloy system, considering all sub‐compositions at a 1% molar resolution, the total number of possible combinations exceeds 4.3 × 10^12^ [54]. Remarkably, techniques such as flame spray pyrolysis [16, 218] and discharge plasma [119] have successfully integrated over 20 elements into a single‐phase solid solution, a feat unachievable via near‐equilibrium methods. These capabilities unlock vast opportunities for creating previously inaccessible materials with unique physicochemical properties, potentially redefining long‐standing principles in materials science. However, with these opportunities come equally significant challenges. The vastness of the possible composition, structure, and property space, encompassing diverse material species, crystal structures, and compositional permutations, introduces unprecedented complexity into the material design. Despite the ability of high‐throughput synthesis to rapidly generate relatively large numbers of material samples, traditional trial‐and‐error experimentation or simple experimental design strategies are ill‐suited to identifying optimal candidates from among millions of possibilities. The key scientific and technological question becomes: how can we efficiently navigate this complex landscape to identify and synthesize truly high‐performance, cost‐effective, and scalable materials tailored for specific applications?

In recent years, advances in AI and machine learning have significantly transformed this landscape, offering data‐driven strategies to accelerate materials discovery [219]. By leveraging existing experimental and computational databases, machine learning models can be trained to uncover complex, non‐linear relationships between material composition, structure, and performance. These models can then be used in two complementary modes: forward design, which predicts material properties from known structures, and inverse design, which proposes promising candidates to achieve targeted properties. When integrated with necessary experimental data, these models enable closed‐loop materials discovery, an iterative process in which an AI/ML based model continuously refines its predictions based on experimental feedback, thus reducing the number of required experiments while increasing the probability of finding high‐performance materials [220]. For example, using a Suzuki coupling reaction to generate a virtual library of 1 million organic semiconductors from 1132 A‐type and 850 B‐type monomers, a closed‐loop workflow combining high‐throughput experiments and AI‐driven inverse design was developed [221]. After two iterative cycles, a hole‐transport material enabling 26.2% power conversion efficiency and over 1000 h of stability (T_80_) in perovskite solar cells was successfully discovered.

In the context of AI for materials discovery, providing a large and diverse database is a fundamental prerequisite for effective AI‐driven screening. However, most current studies are limited to known material systems and existing databases. In contrast, the nearly infinite compositional space enabled by non‐equilibrium synthesis of high‐entropy materials offers a vast and original virtual dataset for AI‐guided screening and optimization. Advanced global optimization algorithms can be employed to rapidly identify promising compositions. These predictions can then be validated through high‐throughput experiments, and the results fed back to further refine the AI models, improving their accuracy and predictive power. Through such closed‐loop iterative cycles between non‐equilibrium, high‐throughput synthesis and AI, it becomes possible to efficiently screen and identify high‐performance materials tailored to specific applications.

Therefore, in the era of AI, high‐entropy nanomaterials synthesized by non‐equilibrium methods hold the potential to transform the landscape of materials research and development. However, to date, non‐equilibrium synthesis methods have not been as extensively explored as traditional wet‐chemical approaches. One major reason is that many non‐equilibrium techniques require specialized and often expensive equipment, in contrast to conventional solution‐based methods, which typically rely on simple glassware and basic heating tools that are readily accessible in most laboratories. This disparity in accessibility has, to some extent, limited the broader development and application of non‐equilibrium synthesis methods. We hope this review highlights the unique advantages and vast potential of non‐equilibrium synthesis methods in integrating immiscible elements and creating metastable or high‐entropy nanomaterials. With their inherent strengths in high‐throughput synthesis and industrial scalability, these methods, when combined with AI‐driven materials discovery, could achieve the rapid and large‐scale advances in high‐performance materials across fields such as catalysis, energy storage, sensing, and biomedicine. This powerful convergence may help usher in a new era of accelerated materials innovation, providing a promising path toward addressing global challenges like energy scarcity, environmental pollution, and climate change.

## Author Contributions

S.L. wrote the manuscript; C.C.D., J.J.U., and M.T.S. revised the manuscript; C.C.D., J.J.U., and M.T.S. jointly supervised this work.

## Conflicts of Interest

The authors declare no conflicts of interest.
